
JOURNAL INFORMATION
==============================
NLM Title Abbreviation: JBRA Assist Reprod
Journal ID: JBRA Assist Reprod
NLM Journal ID: 101684552
Journal ID: jbra

JBRA Assisted Reproduction

ISSN: 1517-5693
EISSN: 1518-0557
Publisher: Brazilian Society of Assisted Reproduction

ARTICLE INFORMATION
==============================
PMCID: PMC6106627
PMID: 30129354
DOI: 10.5935/1518-0557.20180064
Article version: 1
Subjects: Poster Presentations

Abstracts of the 22st Annual Congress of the SBRA, Brasilia/DF, 01-04 August 2018

Publication date: 2018 Jul-Sep
Print publication date: 2018 Jul-Sep
Volume: 22
Issue: 3
First page: 267
Last page: 283
License: This is an Open Access article distributed under the terms of the Creative Commons Attribution License, which permits unrestricted use, distribution, and reproduction in any medium, provided the original work is properly cited.
License URL: https://creativecommons.org/licenses/by/4.0/

==============================

**V.M. Lopes, J.P.B Brasileiro, J.R.C. Lopes, T.S. Lacerda, C.A. da Rocha, S.A. de Oliveira, P.C.M. Pereira**

**Objective:** To compare the results of in vitro fertilization (IVF)/intracytoplasmic sperm injection (ICSI) cycles in women with good prognosis, when they were electively transferred, a blastocyst stage embryo (group 1) versus a blastocyst stage and a mórula stage embryo (group 2).
**Methods:** 35 transfers from January/2014 to January/2018 were included, these occurred in na assisted reproduction center. Selection criteria: Patients in first or second treatment for IVF/ICSI less than 35 years old, with body mass index <30kg/m$^2$ who used their own oocytes with normal ovarian reserve (baseline of antral follicles ≥11) and recipients without age restriction. Cases of severe male fator (spermatic concentration below 1million/ml) were excluded. The transfer was realized on fifth day of embryonic development. Luteal phase support was performed with micronized progesterone 600mg/day vaginally and estradiol 6mg/day orally, started the day after eggs collection. The receivers used 6mg/day of estradiol valerate orally, beginning on first day of menstrual cycle and 600mg/day of micronized progesterone, vaginally, for five days before embryo transfer. Were evaluated: mean age of women under 35 years who used their own oocytes and donors; pregnancy rate (βHCG positive); pregnancy loss rate and ongoing pregnancy rate (fetus alive at 12th week of pregnancy).
**Results:** Between group 1 (n=24) and 2 (n=11), mean age of women under 35 who used their own oocytes were 33(±1.6) and 31(±1.8) years old and for donors were 34(±0.5) and 29(±0) years old, for both groups, respectively. Pregnancy rate was 50% (12/24) for group 1 and 18% (02/11) for group 2 ($p$=0.02). Pregnancy loss rate was 17% (02/12) for group 1 and 50% (01/02) for group 2 ($p$=0.07). Ongoing pregnancy rate was 42% (10/24) for group 1and 9% (01/11) for group 2 ($p$=0.01).
**Conclusion:** The analysis of our data shows that transfer another embryo with a slower development with a blastocyst, with the intention of increase pregnancy rates, can actually worsen the results. There is a worldwide trend about extending embryo culture by fifth or sixth day, then these data should be validated in a larger number of cases. The reason for an embryo with slower development impact on development of another embryo with normal development should be evaluated. We emphasize that the results were extracted from cases with good prognosis and can not be extrapolated to other cases.

## P-02. Amino acids supplementation in culture of cumulus-oocyte complexes: does it matter?

**M.I.R. Kulmann, C. Martello, A. Bos-Mikich, N. Pagnoncelli, G. Frantz, C. Dutra, N. Frantz**

**Objective:** Oviduct and uterine fluids contain significant levels of free amino acids which play important roles in oocyte and embryo metabolism. In fact, studies on different mammalian species have revealed impaired embryo development when embryo culture takes place in media lacking amino acids. Despite the great interest that amino acid supplementation in embryo culture media has received, few studies have adressed the amino acids supplementation in COC or oocyte culture media. In this sense, several fertility centers choose media which are not supplemented with amino acids, commonlly cheaper, for the first steps of IVF. So, does the culture of cumulus-oocyte complexes (COCs) after oocyte pick-up (OPU) in media supplemented with amino acids improve embryonic development and clinical outcomes?
**Methods:** Prospectively randomized study was performed on 1075 COCs retrieved from 89 cycles (87 patients) between September 2017 and December 2017. The rates of maturation, fertilization, good quality at cleavage stage, blastocyst formation, good quality blastocyst and implantation were analyzed. Patients under 40 (26 - 39) years old treated by ICSI with controlled ovarian stimulation were randomly divided into 2 groups. After OPU, COCs were cultured for 3 hours in HTF Medium (Irvine Scientific, USA) or Continuous Single Culture Medium (Irvine Scientific, USA) matching the conditions of No Supplementation (A) and Amino Acids Supplementation (B) respectively. Thereafter, only Continuos Single Culture Medium (Irvine Scientific, USA) was used for oocyte/embryo culture until day-6 in both groups.
**Results:** Groups A and B were similar regarding age (34.1±3.4 vs. 34.7±3.4; $p$=0.3820), BMI (24.2±4.0 vs. 24.0±4.3; $p$=0.8037), Anti-Müllerian Hormone levels (1.7±1.5 vs. 2.1±1.8; $p$=0.2738) and gonadotrophin dose during controlled ovarian stimulation (1648.6±691.9 vs. 1590.3±633.3; $p$=0.6794) respectively. Also, there were no statistical differences between groups A and B concerning number of follicles (16.6±11.9 vs. 16.3±10.3; $p$=0.8697), number of COCs (12.6±9.1 vs. 11.5±10.3; $p$=0.5460), maturation rate (81.3% vs. 83.1%; $p$=0.4734), fertilization rate (75.0% vs. 66.9%; $p$=0.3868), good quality at cleavage stage rate (61.2% vs. 60.5%; $p$=0.8689), blastocyst formation rate (61.9% vs. 57.6%; $p$=0.3074) and good quality blastocyst rate (36.2% vs. 33.3%; $p$=0.6503) respectively. Finally, 22 (50%) group A cycles and 20 (44.4%) group B cycles had fresh embryo transfers, resulting in similar implantation rates between the two groups (A=37.0% vs. B=40.6%; $p$=0.8062).
**Conclusion:** Several studies have argued that the exact composition of media is often not disclosed properly by the manufacturers. Therefore, the absence of amino acids in HTF Medium is questionable. Moreover, new data related to delivery rates and outcomes from frozen embryos need to be taken into account. Our study suggests that media without amino acids supplementation can be used harmlessly for COCs culture, which highlights the significant physiological role of cumulus cells preventing homeostatic stress in vitro. To our knowledge, this is the first study to assess the impact of amino acids supplementation in human COCs culture.

### P-03. Quantitative and qualitative analysis of microorganisms in an Assisted Reproduction laboratory

**K.Q. Poletto[1], Y.A.R. de Lima[1], D.F. Rezende[1], E.C. de Castro[1], M.S. Approbato[1]**

**Objective:** To perform a quantitative and qualitative analysis of environmental microorganisms present in an assisted reproduction laboratory.
**Methods:** Air samples were collected from three sites at three time points: the in vitro fertilization laboratory bench top and from two embryo incubators (incubator-1 and -2). The air collection was performed monthly for three months by exposing TSA Agar (Trypticase soy agar) and SDA Agar (Sabouraud agar) plates, for 4 hours. Plates were incubated at 37 °C for 48 hours, sealed and incubated for another 48 hours in order to perform the Colony Forming Units (CFU) counting. Microorganisms grown were identified through biochemical and enzymatic tests.
**Results:** No significant variation in the CFU counting was observed during this time period at the three sites ($p$=0.9522). Within the incubator-1 it was observed four CFUs at the first collection, one CFU at the second and no CFU at the third one (mean=1.67; SD=±2.1). Regarding the air collected in the incubator-2, a total of two CFUs were identified at the first month, one CFU at the second and one CFU at the third one (mean=1.33; SD=±0.6). Regarding the environmental air sampled in the bench top plate, no CFU was identified at the first month, five CFUs at the second one and one CFU at the third one (mean=2.00; SD=±2.6). The following microorganisms were isolated: Enterococcus sp, Staphylococcus saprophyticus, Staphylococcus epidermides, gram positive bacilli and hyaline septated filamentous fungus.
**Conclusion:** In vitro fertilization laboratories do not routinely monitor the microbial counting outside the incubator environment. Therefore, the laboratory staff may be unaware of the microorganisms density. These pathogens can contaminate the embryo culture media plates, which directly interfere with the rates of gestation and birth. An environmental microbiological monitoring is necessary to define the appropriate time for the exchange of the air filtration system, in order to achieve a cleaner air for the embryo cultures.

### P-04. Aneuploidy screening data in Brazilian population - 4.5 years of PGS/PGT-A in IVF-lab routine

**I. Reis[1], G. Romanenghi[1], B. Barros[1], A.R. Lorenzon[1], M.B. Chehin[1]**

**Objective:** Aneuploidy is the main genetic factor associated with infertility. Preimplantation genetic screening (PGS), currently named Preimplantation genetic testing for aneuploidy (PGT-A) has been routinely used in IVF centers worldwide to help the selection of the best embryo for transfer. Our objective is to compile data from 4,5 years of aneuploidy screening and compare our data with other international centers.
**Methods:** This is a general data profile from 2143 cycles and 5084 biopsied embryos (trophectoderm biopsy, blastocyst stage) from January 2014 until July 2017, for aneuploidy screening (PGS/PGT-A) from patients undergoing IVF cycles at Huntington Medicina Reprodutiva (São Paulo, Brazil). Genetic indication and oocyte donation were not considered for this purpose. Embryo biopsy platforms used for diagnostic were CGH-a (2014-2015) and NGS (2015-2017). Aneuploidy were stratified by age (<38 years old and ≥38 years old) and by type - single, double or multiple chromosomes aneuploidy. Single chromosome aneuploidy also considered the chromosome number (1-23, X, Y) frequency.
**Results:** In total, 2213 embryos (843 cycles) were biopsied from women between 24-37 years old and 2871 embryos (1300 cycles) from women between 38-45 years old. From the first group (24-37 years old), 1173 embryos (53%) were diagnostic as euploid and 991 (48%) as aneuploid. Women's average age between euploid and aneuploid embryos were similar (34,2 vs 34,7 years old respectively) in this group. Considering aneuploid embryos (n=991), 68% were classified as single chromosome aneuploidy, 18% as double and 14% as multiple chromosome aneuploidy. In the single chromosome aneuploidy, the most frequently were chromosomes 22 and 16 (14% each), followed by chromosome 21 (8%) and 15 (7%). In the second group (38-45 years old), 830 embryos were diagnostic as euploid (29%) and 1979 (69%) as aneuploid. Women's average age between euploid and aneuploid embryos were also similar (39,8 vs 40,5 years old respectively) in this group. Considering the aneuploid embryos (n=1979), 48% were classified as single chromosome aneuploidy, 28% as double and 24% as multiple chromosome aneuploidy. In the single chromosome aneuploidy, the most frequently was on chromosomes 22 (15%), followed by chromosome 16 (13%), chromosome 21 (10%) and chromosomes 15 and 19 (7% each). All other chromosomes for single aneuploidy, considering all ages had an incidence of 1 to 5%.
**Conclusion:** This aneuploidy screening is important to verify if the aneuploidy rate in our population is consistent with other groups. In two studies published by Franasiak and collaborators, 2014 (USA), with 15.169 biopsied embryos, the rates of aneuploidy in women between 26 until 39 years old is 24-52.9% and between 40 and 43 years old is 58.2-83.4%. In our practice, women between 24-37 years old the aneuploidy rate is 48% and between 38-45 years old is 69%. They also detected an increase in more complex aneuploidies (double or multiple chromosomes) with older maternal age, and the most frequently observed aneuploidy was seen for chromosomes 13, 15, 16, 18, 19, 21, and 22, independently of women's age. In general, our data screening is consistent with other IVF centers worldwide.

### P-05. Anogenital distance is associated with semen quality and serum reproductive hormones in Brazilian young men

**M.S. Procópio[1], D.C. Borges[1], C.G. Fernandes[1], M.L. dos Santos[1], R.A. Leite[1], S.M.S.N. Lacerda[1], L.R. de França[1], A.B. Reis[2]**

**Objective:** The purpose of this investigation was to examinate the possible association of anogenital distance (AGD) with semen parameters and serum reproductive hormone levels in brazilian young men.
**Methods:** In this study, two variants of AGD (AGDap and AGDas) were measured in 235 brazilian university students from Belo Horizonte metropolitan area. Serum levels of testosterone, estradiol, progesterone, luteinizing hormone, follicle-stimulating hormone, sex hormone binding globulin (SHBG); and semen quality outcomes, including semen volume, sperm concentration, total sperm number, sperm progressive motility, non progressive motility, and total motility were assessed. The associations between AGD and semen parameters/reproductive hormones levels were analyzed using univariate regression analysis.
**Results:** AGDas was associated with semen parameters and AGDap with hormonal serum levels. In the nonparametric correlation analysis, AGDas were correlated with sperm non progressive motility (r=0.23; $p$=0.004); total motility (r=0.17; $p$=0.01); sperm concentration (r=0.13; $p$=0.04) and total sperm count (r=0.15; $p$=0.02). AGDap only showed association with SHBG (r=-0.28; $p$=0.0007). However, body mass index (BMI) also significantly correlated with AGDas (r=0.45; $p$<0.0001), AGDap (r=0.14; $p$<0.0001) and SHBG serum levels (r=-0.35; $p$<0.0001).
**Conclusion:** Our results support previous studies where AGD is associated with male semen quality and serum reproductives hormones.

### P-06. Evaluation of the influence of the number of retrieved oocytes on the success of ICSI

**J. Polisseni[1], C.M. de Assunção[1], F. Polisseni[1], J.V. Rosa[1], L.M. Coutinho[1]**

[1]Clínica Nidus Medicina Reprodutiva.

**Objective:** To evaluate the influence of the number of retrieved oocytes, inpatients submitted to the Intracytoplasmic Sperm Injection (ICSI) technique, after oocyte collection, on the success of ICSI.
**Methods:** A retrospective study was performed at Clinical of Reproductive Medicine in Zona of Mata Mineira by selection medical records. The study included 426 women, mean age 35.42±4.07, submitted to the ICSI in the period 2016 to 2018 and who obtained one or more oocytes at metaphase II stage, after oocyte collection. Ranged age was 18 to 44 years. Ovarian stimulation was performed with Gonadotropin-releasing hormone antagonists. The variables analyzed were: number of oocytes retrieved after oocyte collection, fertilization rate, cleavage rate, blastocyst rate, total pregnancy rate and clinical pregnancy rate. To analyze the results, the patients were divided into 4 groups: Group I - 1 to 5 oocytes collected (n=106); Group II - 6 to 10 oocytes collected (n=128); Group III - 11 to 15 oocytes collected (n=99); and group 4 - greater than 16 oocytes captured (n=93). The variables were analyzed by the chi-square test, using GraphPad Prism, version 7.04 ($p$<0.05).
**Results:** The patients in the four groups showed similar rate of fertilization (84.15%, 87.44%, 86.12% and 88.94%) and cleavage (93.12%, 94.84%, 94.63% 93.78%), respectively in groups I, II, III and IV. However, the pacients of group I had the lowest blastocyst rate (11.28% versus 36.95%, 53.77% 60.20%), total pregnancy (28.30% versus 48.44%, 48.44) and clinic pregnancy (21.70% versus, 45.31%, 44.44% and 50.54%), ($p$<0.05), respectively, in groups I, II, III and IV ($p$<0.05), when compared to the other three groups. Groups II, III and IV did not differ in the parameters of total and clinical pregnancy rate ($p$>0.05), although groups III and IV had a higher blastocyst rate compared to the other two groups ($p$<0.05).
**Conclusion:** The success of ICSI is related to the number of oocytes collected in patients submitted to assisted reproduction techniques. Women obtained six oocytes or more per ICSI cycle (groups II, III and IV) showed better results. This fact seems related to the possibility of selection of embryo for transfer, since there is evidence of a positive association between higher blastocyst rates and better rates of total and clinical pregnancy.

### P-07. Evaluation of seminal parameters after freezing in two types of Brazilian cryo-probes: with or without egg yolk low-density lipoprotein

**R.L. Bossi[1], B.C. Villa[2], M.A.C. Sampaio[2], P.P. França[1], D.M. Alvarenga[1], S. Geber[1]**

**Objective:** Cryopreservation is an important alternative for reproductive medicine, since it allows the storage of surplus embryos, oocytes and spermatozoa for future treatments. Cryoprotectants are chemicals that prevent cell damage by minimizing intracellular ice formation and osmotic stress. Several techniques for sperm freezing have been described using different cryoprotectants. However, poor recovery rates of viable sperm after cryopreservation are responsible for unsatisfactory samples for assisted reproduction treatments. In order to improve results, reduce costs and eliminate the animal component of the cryoprotectant, the present study aims to compare two sperm freezing media used in normal semen samples.
**Methods:** A prospective observational study from February to December 2017 was performed in a private human assisted reproduction clinic in Brazil. All patients signed informed consent form. Each sperm samples was frozen in two different cryopreservation media: an aliquot with Ingá Sperm Freezing without low-density lipoprotein (LDL) of the egg yolk (Ingamed-Brazil) and another aliquot with Ingá Sperm Freezing with LDL from egg yolk (Ingamed-Brazil). The samples were frozen/thawed according to the manufacturer's instructions. After thawing, all samples were evaluated for motility, and morphology. Data were analyzed by Student t-test and McNemar's Qui-Square test.
**Results:** A total of 47 sperm samples were included in the study. The mean age of the patients was 37.7±6.3 ranging from 24 to 55 years. Sexual abstinence was 4.5

days±4.5. Mean seminal volume was 3.6±1.4ml, (1 to 7mL). When we compared both groups of cryoprotectant we observed that samples frozen/thawed with Ingá Sperm Freezing without yolk, had a significantly higher sperm motility (23.9±19.5 vs 13.8±13.3, $p<0.0001$). (Figure 1). The same was observed for normal morphology, i.e., better results with Ingá Sperm Freezing without yolk (9.5±5.9 vs 6.0±4.0, $p<0.0001$). Among the morphological defects observed, flagella was significantly more compromised in the Ingá Sperm Freezing medium with yolk (12.5±5.4 vs 10.8±5.3 $p=0.03$).
**Conclusion:** The results showed that Ingá Sperm Freezing medium without yolk gives better results of sperm motility and morphology when compared to the medium with Ingá Sperm Freezing with yolk.

### P-08. Clinical outcome of in vitro maturation treatment in a series of patients with polycystic ovaries and polycystic ovaries syndrome

**A. Bos-Mikich[1], M.I.R. Kulmann[2], N.P. de Oliveira[2], C. Gross[2], B. Toson[2], M. Ferreira[2], N. Frantz[2]**

**Objective:** IVM represents a patient-friendly, lower-cost fertility treatment, as the patients do not need high-doses of gonadotropins for ovarian stimulation. However, these benefits have not been enough to motivate clinicians to recommend and to use the methodology. When the technology is specifically used for patients with polycystic ovaries syndrome (PCOS) or polycystic-like ovaries (PCO-like), IVM have demonstrated results similar to classic superovulation cycles. The objective of the present work is to report the outcomes of 34 IVM cycles performed between 2013 and 2018, at Nilo Frantz Human Reproduction Center in patients with PCOS or PCO-like.
**Methods:** Thirthy four patients with PCOS or PCO-like (according to the Rotterdam criteria, 2003) were recommended IVM treatment. No gonadrotropin was used for follicular growth. Spontaneously menstruating patients underwent a baseline ultrassound scan at Day-2 of their menstrual cycle for antral follicle count; in anovulatory patients, a withdrawal bleeding was induced by the use of dihidrogesterona (10mg/5 days). When patients presented a trilaminar endometrium of 6mm, 10.000 UI of hCG (Choriomon®) was administered. Thirty six hours post-hCG follicular puncture was performed with a 19-Gauge needle (Cook) at an aspiration pressure of 75-80 mmHg. Follicular punctures were guided by multifrequency vaginal probe (5 a 9 mHz; Ultrasonix OP, Sonix). The oocyte-cumulus complexes (OCCs) were retrieved and cultured in pre-maturation medium for three hours (LAG, Medicult, Origio), before IVM culture for 32 hours (Medicult, Origio, suplemented with FSH e hCG). After denuding, mature oocytes (at metaphase II) were inseminated by ICSI. Zygotes were cultured until day 3 or 5. Ten days after embryo transfer, βhCG-test was performed. To confirm clinical gestation, the ultrassound exam was carried out two weeks later.
**Results:** The mean age of the patients was 32.14 years (between 23 and 36) and their mean BMI was 24.93 (between 17.4 and 36). Mean serum AMH level was 11.62 ng/ml (between 5.94 and 32.30) and the mean endometrial thickness was 6.2 mm (between 3.0 and 8.0mm). In vitro maturation and fertilization rates were 70.67% and 71.26%, respectively. An average of 2.47 embryos were transferred per patient (one to three/patient). Overall embryo transfers yielded a positive βhCG test in 40% of the patients and 36.66% clinical gestation per transfer. The total birth rate was 30% per transfer. Pregnancies were uneventful and no birth defects were detected.
**Conclusion:** The outcomes in 34 patients with polycystic ovaries or polycystic ovaries syndrome demonstrate that IVM is a valid option for fertility treatment in this group of women. Pregnancy and birth rates are very similar to those obtained after classical ovarian stimulation cycles. These data motivate us to continue using IVM and to recommend the use of this methodology among the assisted reproduction technologies currently in practice.

### P-09. Correlation between number full-term pregnancies after ICSI treatment and day of embryo transfer

**J. Polisseni[1], C.M. de Assunção[1], F. Polisseni[1], J.V. Rosa[1], L.M. Coutinho[1]**

*[1]Clínica Nidus Medicina Reprodutiva.*

**Objective:** To correlate the number full-term pregnancies of patients submitted to the Intracytoplasmic Sperm Injection (ICSI) technique, with day of embryo transfer: day two/three (cleavage stage) and day five/six (blastocyst stage).
**Methods:** A retrospective study was performed at Clinical of Reproductive Medicine in Zona of Mata Mineira by selection medical records. The study included 649 patients, with a mean age of 35.22±4.39, from 2014 to 2017 and transferred fresh embryos, after ICSI, on days two, three, five or six of embryo culture. Ranged age of patients was 18 to 45 years. The variables analyzed were: total, clinical and biochemical pregnancy rate, abortion rate and number full-term pregnancies. To analyze the data, the patients were divided into two groups: Group I - embryo transfer D2/D3 (n=349); and Group II - embryo transfer D5/D6 (n=300). The variables were analyzed by the chi-square test, using GraphPad Prism, version 7.04, ($p<0.05$).
**Results:** Group II patients had a higher total pregnancy rate (47.67% versus 37.54%) when compared to group I ($p<0.05$). However, the clinical pregnancy rate (34.38% and 38.33%) and full-term pregnancies (70.83% and 80.87%), respectively, groups I and II, did not showed statistical differences ($p>0,05$), although they are numerically larger in group II. Biochemical pregnancy rates were higher in group II (19.84% versus 8.40%) when compared to group I ($p<0.05$). The abortion rate was not different between the groups (26.67% and 17.39%), respectively groups I and II ($p>0.05$), although it presented a higher number in group I.
**Conclusion:** For better embryo selection embryos are frequently transferred in the blastocyst stage. However, the study showed that the number of full-term pregnancies does not appear to be correlated with the day of embryo transfer. Despite patients that transfered in the blastocyst stage showed greater number of total pregnancies, the number of clinical and full term pregnancies did not had statistical difference between the groups, although they are numerically greater in group II.

### P-10. Clinical data of patients who cryopreserved spermatozoa at a private clinic in Curitiba/PR due to future gonadotoxic treatments

**D. Scaraboto[1], L.J.R. Centa[1], I.T. Saruhashi[1]**

**Objective:** Cryopreservation is still the first and best option for patients who will undergo chemo or radiotherapy treatments. Our goal with this study is to show the profile of oncological patients who sought cryopreservation of spermatozoa in our bank in the last 5 years, stratifying them by type of cancer.
**Methods:** 105 male cancer patients were submitted to cryopreservation of semen in the ANDROLAB semen bank during a 5-year and 4-month period (October 2012 to February 2018) for being submitted to gonadotoxic treatments, such as chemotherapy and radiotherapy or even orchiectomy. All patients who freeze semen at the ANDROLAB bank are seen by a qualified professional before freezing. The type of cancer was documented according to information provided by the patient and/or the referring clinician. The semen analysis followed the pattern established by the World Health Organization in its 2010 "Laboratory Manual for the examination and processing of human semen". Azoospermic patients on the day of cryopreservation were excluded from the study, as were those who did not want to or could not report on their type of cancer.
**Results:** The sperm concentration per mL pre-cryopreservation was 58.7 million and the motility (spermatozoa with progressive movement) was 41.6. Testicular cancer was diagnosed in 37 patients (31%), lymphomas (Hodgkin or non-Hodgkin) in 15 patients (13%) and leukemia in 8 patients (6.7%). Other cancers were identified in fewer numbers, in 45 (42.8%) patients. Stratifying by type of cancer, we notice that the concentration of spermatozoa in patients with testicular cancer was 41.6 million per mL; 20.4 million per mL for lymphoma patients and 88.2 million per mL for leukemia patients. The mean motility in patients with testicular cancer was 40.2% spermatozoa with progressive motility; in lymphoma patients, it was 18% and in leukemia patients it was 34.4%. Four percent of the patients sought sample thawing for gestation purposes. 29% returned within the 5-year period with satisfactory results of seminal analysis after the gonadotoxic treatment and opted to discard the cryopreserved material. The mean post-thawing motility was 22.1%.
**Conclusion:** Data collection from the semen bank of this private clinic showed that patients diagnosed with Hodgkin or non-Hodgkin lymphomas had the lowest pre-freezing semen quality compared to the average. Most cancer patients who seek cryopreservation of their sperm are affected by testicular tumors. This may indicate a trend already known in the clinical practice of oncology that indicate few patients undergoing semen cryopreservation, except for the cases in which the neoplasia itself affects sperm production. Therefore, there is a need to educate oncologists on the indication of semen freezing according to the chemotherapy and the dose applied, and not just according to tumor location.

### P-11. Delayed intracytoplasmic sperm injection: is it worth it?

**D.P.A.F. Braga[1,2], C.A.R. Iaconelli[1], C.C.V. Brogilato[1], B.F. Zanetti[1,2], A.S. Setti[1,2], A. Iaconelli Jr.[1,2], E. Borges Jr.[1,2], R.C. Ferreira[1]**

**Objective:** To study delayed intracytoplasmic sperm injection (ICSI) cycles with oocytes matured the day after ovarian puncture.
**Methods:** Data was obtained from 486 ICSI cycles performed in a private university-affiliated in vitro fertilization center between Jan/2000 and Dec/2017. The outcomes of cycles with retrieval of only immature oocytes (metaphase I or germinal vesicle stage) injected the day after ovum pick-up at metaphase II (MII) stage (Group A, n=40) were compared to cycles in which ≤2 oocytes were retrieved, but at least one MII oocyte was injected the same day of ovum pick-up (Group B, n=446), by Mann-Whitney test and Chi-Squared.
**Results:** Mean maternal age (A: 38.25±4.45 vs. B: 39.61±4.43, $p$=0.052), number of follicles (A: 5.23±7.22 vs. B: 3.30±1.68, $p$=0.969), retrieved oocytes (A: 2.20±1.83 vs. B: 1.58±0.49, $p$=0.638) and injected oocytes (A: 1.93±1.74 vs. B: 1.44±0.50, $p$=0.932) were similar between groups. Normal fertilization was lower in group A (A: 0.90±1.32 vs. B: 0.96±0.67, $p$=0.017), while the number of non-cleaved embryos was higher (A: 0.30±0.82 vs. B: 0.02±0.16, $p$<0.001). The percentage of cycles with fresh embryo transfer (A: 55.0% vs. B: 61.7%, $p$=0.679), number of transferred embryos (A:0.80±0.98 vs. B:0.78±0.71, $p$=0.523), clinical pregnancy rate (A:9.1% vs. B:14.2%, $p$=0.554), ongoing pregnancy rate (A:4.5% vs. B:10.2%; $p$=0.426) and miscarriage (A: 4.5% vs. B: 4.0%, $p$=0.904) were similar between groups.
**Conclusion:** Embryos from delayed ICSI have the same development potential as those derived from mature oocytes of poor responder patients.

### P-12. Difficulties in homoaffective couples' IVF

**A.K. Bartmann, L.F.I. da Silva, A.L.C. Franco, K.T. Moriwake, L.F. Brioni**

**Objective:** Reproductive desire considered a natural consequence of the union of most heterosexual couples has also been experienced by homosexual couples who are increasingly seeking assisted reproductive techniques to achieve it. The legalization of same-sex marriage in many countries of the world has brought about the need to broaden the discussion about the reproductive issues of this population. Homosexual couples face obstacles of various natures: legal, for lack of specific laws; cultural and social, since there is still much prejudice and lack of knowledge on the subject; because there is no compatibility in natural reproduction between two persons of the same sex; or even financial. The objective of this article is to discuss the difficulties faced by homoaffective couples with procreative desire.

**Methods:** Literature review using databases: Pubmed, Medline, Google Scholar and SciELO.
**Results:** During the study we found that couples composed of two women opted for assisted reproduction more than couples composed of two men. This is probably due to the difficulty men face in donating oocytes and replacing the uterus. In addition, we note a lack of global consensus on assisted reproduction in the specific case of homosexual couples due to various existing ethical and legal gaps.

### P-13. Does endometrioma surgery affect pregnancy rates in women with intestinal endometriosis?

**M.M. Carneiro, L.C.V. Amorim, I. de Ávila, L.M.P. Costa, L.G.R. Machado, M.C.F. Ferreira**

**Objective:** To study the impact of endometrioma on the fertility of women with intestinal DE undergoing surgical treatment with a multidisciplinary team of endometriosis.
**Methods:** A retrospective cohort study performed at the Biocor hospital with women who underwent surgical treatment for intestinal DE treatment from May 2007 to May 2016. Participation in this study was entirely voluntary and the protocol approved by the internal review board (CAAEE: 41327014.5.0000.5149). A total of 212 surgeries were performed, of which 106 were DE with intestinal involvement 60 of them attempted to become pregnant after surgery. Endometrioma was found and operated in 41 of them. The final outcome analyzed was the pregnancy rate associated with endometrioma surgery. The cumulative pregnancy rate was calculated and $p<0.005$ was considered significant.
**Results:** Of the 60 patients with DE who desired pregnancy, 41 (68.3%) underwent surgical treatment of the endometrioma. Of these 41 patients, 18 became pregnant with a general pregnancy rate of 64.3%. No negative association between the presence of endometrioma and the occurrence of pregnancy was found. ($p=0.586$). Of the 41 patients who wished to become pregnant, 16 underwent IVF, and 6 of them became pregnant. The success rate of IVF treatment was 37.5% in this group. Of the 25 patients who were not submitted to IVF, 12 (48%) had spontaneous pregnancies. The mean age of the patients who became pregnant was 33.6 years in the IVF group and 31.6 years in the spontaneous group. The time elapsed between surgery and pregnancy was 11.7 months in the IVF group and 6.5 months in the spontaneous group.
**Conclusion:** Although the literature points out that the presence of endometrioma and its exeresis may have a negative impact on the pregnancy rate, in this group of patients studied no such effect was observed. The surgical approach of DE and endometrioma in a multidisciplinary center specialized in endometriosis is vital for fertility preservation of fertility and maintenance of future pregnancy chances.

### P-14. Endometriosis III and IV as a risk factor for tubal obstruction among infertile women

**F.C. Approbato[1], M.S. Approbato[1], D.F. Rezende[1], M.S. Ramos[1], E.N. Santos[1], M.Z.P. Brito[1], T.M. Silva[1], M.C.S. Maia[1], Y.A.R. Lima[1], A.V.S. Moraes[1], E.C. Castro[1], K.Q.P. Rosilho[1], R.S. Sasaki[1], M.C.F. Finotti[1]**

**Objective:** A previous study performed among infertile women with tubal obstruction identified a relative risk of 2.5 for Chlamydia trachomatis seropositivity. However, endometriosis may be also related with increased risk. This study aimed to evaluate the risk of tubal obstruction associated with endometriosis III/IV among women submitted to reproductive assisted procedures.
**Methods:** A case-control study was performed among 144 women with and without tubal obstruction. We calculated the odds ratio with 95% CI of association with endometriosis III/IV and tubal obstruction. Calculations were performed using SPSS package v.17.0.
**Results:** Media of age was 33.7 years (4.76 SD) and media of fertility duration time was 66.7 month (120.6 SD). Endometriosis total prevalence was 20/144 (13%). Among 144 women, the risk group with tubal obstruction and endometriosis III/IV comprised 7out of 20 (35%) compared with the group without risk that comprised 22 out of 124 (17%). The X2 test was 3.19 with a p value of 0.07. Odds ratio (OR) was 2.5 (95% CI: 0.647-9.639).
**Conclusion:** Although OD was 2.5, there was no significant difference among groups with and without endometriosis III/IV. Further studies are needed to increase the population number.

### P-15. Tubal factor etiologies in infertile patients from a public assisted reproductive service

**D.F. Rezende[1], Y.A.R. de Lima[1], K.Q. Poletto[1], M.S. Approbato[1], E.C. de Castro[1], A.V.S. Moraes[1]**

**Objective:** Determine the etiologies of the tubal factor in infertile patients of a public assisted reproductive service.
**Methods:** This is a cross-sectional retrospective study with a quantitative approach data from patients seen at the Human Reproduction Laboratory of the Clinical Hospital of the Federal University of Goiás, and the information was taken from the Sisfert 2.0 database. Included in the study were all patients attended at the service, from August 2002 to March 2018, aged between 21 and 40 years, who had a complete record in the database and who had undergone hysterosalpingography to evaluate tubal permeability. The prevalence of tubal alterations was evaluated in patients attending the respective infertility clinic, with determination of the etiologies associated with the tubal factor, using a significance level of 5% ($p<0.05$).

**Results:** The initial n sample was 1784 patients and the final n sample was 1554. Two hundred and thirty patients were excluded from the study because they were older than 40 years or presented incomplete data in the database. The mean age in the group studied was 34.3 years (SD±4.2). The prevalence of tubal alterations in ambulatory patients was 27.79% (n=432, 95% CI, 26-30%). Of these, 215 (49.8%, 95% CI, 45-54%) had bilateral tubal ligation, 115 (26.6%, 95% CI, 23-30%) presented tubal occlusion (unilateral or bilateral), 17 (Salpingostomy or salpingectomy), 10 (2.4%, 95% CI, 1 to 4%) presented hydrosalpinge, 4 patients (0%, 95% CI, 2 to 6%) reported a history of tubal ectopic gestation with surgical treatment (95% CI, 0.01 to 1.0%) with tubal alterations had a history of pelvic inflammatory disease and 71 women (16.4%, 95% CI, 13-20%) were included in other causes (pelvic adhesions, tubal reanastomosis without success and agenesis of the uterine tube, either unilateral or bilateral).

**Conclusion:** The determination of tubal permeability is a fundamental part of the investigation of infertile couples since the tubal factor can represent 20 to 35% of the causes of infertility, coinciding with the prevalence found in this study, which was 27.79%. The tubal evaluation in infertile patients is fundamental to the conducts that must be taken in the therapeutic programming. Although hysterosalpingography, which has high specificity and low sensitivity, has been used in this study for tubal evaluation, it is known that there is no diagnostic method that can guarantee absolute accuracy to the tubal permeability. In this study, it was observed that the main cause of tubal alteration was bilateral tubal ligation, performed as a surgical sterilization method, representing approximately 50% of all tubal factor etiologies. Therefore, the high rate of repentance of this contraceptive method is observed, with repercussions on the reproductive health of these patients.

### P-16. Expression of the Luteinizing Hormone Receptor and its isoforms in animal model for Poor Responder Phenotype

**S. Wohlres-Viana, E.K.N. Arashiro, L.G.B. Siqueira, M.A. Machado, J.H.M. Viana**

**Objective:** The aim of this study was to evaluate the relationship between the expression of Lhr (LHCGR) and its isoforms and response to exogenous ovarian superstimulation, using heifers characterized as good (10.3±1.2 ova/flush, N=5) or poor (1.1±0.3 ova/flush, N=5) responders to superovulation as experimental model.

**Methods:** Granulosa cells were collected from 8 mm follicles growing in either a non-stimulated follicular wave (NS, control) or in the fourth day of superovulation (SOV) induced with 200 IU FSHp. The recovered follicular fluid was centrifuged and the cells recovered kept in RNA Later. Total RNA extraction was performed, and RNA samples were quantified and reverse transcribed using commercial kits. cDNAs samples were amplified by real time PCR, using a non-selective Lhr primer (total Lhr) and four sets of isoforms primers (S1, S10, S10+11, and S11). Analyses were performed using the REST software. Expression values are shown as mean ± SEM.

**Results:** Under physiological conditions (NS), poor responders showed a higher expression of total Lhr (4.9±1.7, $p<0.01$), but also of isoforms S10, S11 and S10+11, when compared to good responders. In both phenotypes, SOV down-regulated total Lhr expression (-0.5±0.2 and -0.9±0.0 for good and poor responders, respectively; $p<0.05$). However, in poor responders SOV up-regulated the S10+11 isoform (1.6±0.8, $p<0.01$), and the expression of S10, S11, and S1 were higher when compared to good responders under the same stimulus.

**Conclusion:** Down-regulation in the expression of total Lhr, and a higher expression of their inactive isoforms, may have compromised follicle development in the poor responder phenotype.

### P-17. Impact of cryopreservation of spermatozoa from PESA and TESE on the prognosis of ICSI. Outcomes from a multicenter study

**T.R. Pereira[1], V.M. Lopes[1], J.P.B. Brasileiro[1], N.Z. Tierno[1], T.S. Lacerda[1], C.A. da Rocha[1], J.J.M. Maciel[2]**

**Objective:** To evaluate the results of couples undergoing in vitro fertilization (IVF) cycles with intracytoplasmic sperm injection (ICSI) using frozen PESA/TESE spermatozoa (group 1) and fresh PESA/TESE spermatozoa (group 2).

**Methods:** We analyzed all treatment cycles within the scope, from January 2014 to January 2018 in two assisted reproduction centers. Spermatozoa collected by PESA/TESE were evaluated by 400-x microscopy to determine the presence of spermatozoa. The samples were then frozen, using YOUK BUFFER (Irvine) for later use (group 1), or fresh use (group 2). The thawed or fresh samples were processed using the different density gradient technique (200µl for each gradient) and washed in culture medium buffered and subjected to ICSI. Selection criteria: women aged up to 40 years, undergoing IVF/ICSI treatment who used their own eggs, with normal ovarian reserve (baseline ultrasonography with antral follicle number ≥11) and recipient with no age restriction. The transfer was performed on the fifth day of embryonic development. The luteal phase support was performed with micronized progesterone 600mg/day via vaginal and estradiol 6mg/day orally. The following were evaluated: mean age of women, fertilized/cleaved eggs, blastocyst, transferred and frozen embryos. In addition to the rates of: gestation (βHCG positive), gestational loss and evolutionary pregnancy (fetus alive at the 12th week of gestation). The t-test and Fisher's exact test were used to compare the results of means and rates, respectively.

**Results:** Between groups 1 (n=29) and 2 (n=16) respectively, the mean age of the women was 34(±6.2) x 37(±3.2) ($p=0.042$). The results of the averages found for groups 1 and 2, respectively, were: 5.93(±2.3) x 7.31(±2.5) ($p=0.067$) fertilized/cleaved eggs, 1.89(±0.7) x 2.43(±0.8)

(*p*=0.01), blastocysts; 1.89(±0.5) x 2.31(±0.5) (*p*=0.0071) embryos transferred, 1.38 (±1.5) x 2.18 (±1.7) (*p*=0.01) frozen embryos. The pregnancy rate, gestational loss rate and evolving pregnancy rate were, respectively, for groups 1 and 2 were: 51.7%(15/29) and 56% (09/16) (*p*=0.737), 27% (04/15) and 11% (n=01/09) (*p*=0.2298), 38% (11/29) and 50% (08/16) (*p*=0.2404).
**Conclusion:** There was no significant difference in the rates of fertilization/cleavage, gestation and abortion when fresh spermatozoa was compared to the use of frozen sperm from PESA and TESE. There is a trend towards the use of fresh spermatozoa in these cases; however, this study did not show significant differences in the ongoing gestation rates. It is worth mentioning that the use of frozen spermatozoa can be of great relevance, since it tends to improve safety margins for the cycle, since spermatozoa are preserved prior to treatment, reducing the risk of no spermatozoa available on the day of collection.

### P-18. Incidence of acquired thrombophilia in Assisted Reproduction

**E. Araújo Filho[1], L.P. de Araújo[1], B.R. de Marchi[1], C.L. Fácio[1], L.A. Machado-Paula[1], L.F. Previato[1]**

**Objective:** Investigate the incidence of acquired thrombophilia (antiphospholipid antibodies) in patients that underwent assisted reproduction (ICSI) with ≥2 IVF failures and/or a history of thromboembolism, miscarriage or adverse complications in previous pregnancies.
**Methods:** This is a retrospective, observational study in which 400 cases of Assisted Reproduction (ICSI) (January 2011 to December 2017) were included, but complete data were 293 cases of ICSI and 37 cases of embryo thawing. The age of women ranged from 21 to 45 years. Inclusion criteria were: ≥2 pregnancy failures in IVF; previous abortion, history of thyroiditis; history of thromboembolism or adverse complications in previous pregnancies. Exams were requested for all patients: anticardiolipin antibody, lupus anticoagulant, antiphosphatidyl serine, antiphosphatidyl ethanolamine, anti thyroperoxidase and anti-thyroglobulin and FAN.
**Results:** Of the 293 ICSI cases included in this study, in 93 the cause was tubal factor (31.7%), 62 were endometriosis (21.1%), 54 were male factor (18.4%), 2 cases of homoaffective union (0, 68%) and 25 insemination failures (8.5%) and 2 were PGD (0.68%). Of the 293 ICSI cases, 233 (79.5%) had antiphosphatidyl serine and/or antiphosphatidyl ethanolamine greater than 15.0 ng/dl (IgG, IgM). There was no case of positive lupus anticoagulant. FAN ≥1/160 was observed in 19 (9.8%) of 293 cases of ICSI and anticardiolipin antibody (IgG and IgM) ≥15.0 ng/dl were found in 7 cases (2.4%).
**Conclusion:** Of the antiphospholipids investigated, the anticardiolipin antibody is less frequently found. With these results we suggest the search for other antiphospholipids such as antiphosphatidyl serine. In view of the relationship between immunology and embryo implantation, we suggest the immunological investigation in couples submitted to IVF before starting treatment. Prospective and randomized studies comparing treat versus non-treat the presence of these antibodies, evaluating implantation rate, pregnancy and abortion should be performed to answer many questions about this subject.

### P-19. Influence of endometrial type on success in Assisted Reproduction Techniques

**J. Polisseni, C.M. de Assunção, F. Polisseni, J.V. Rosa, L.M. Coutinho, V.O. Rodrigues**

**Objective:** To correlate, in a descriptive form, type of endometrium, regarding the ultrasonographic aspect, and success rate of patients submitted to Intracytoplasmic Sperm Injection (ICSI).
**Methods:** Retrospective study was performed at Clinical of Reproductive Medicine in Zona of Mata Mineira by selection medical records to fresh ICSI cycles from 01/2017 to 04/2018. 286 cycles of IVF were performed at this time, but only 95 patients had information on the type of endometrial in their medical records. The patients were divided into 3 groups according to the aspect of the endometrium on ultrasound on the day of hCG: types I, II and III. The data evaluated were: mean age, ovarian stimulation protocol used for ICSI, total pregnancy, clinical and gestational rates in progress, biochemical pregnancy rates and abortion. Endometrial evaluation was performed by the same professional to minimize possible errors. Results were compared by proportion between groups.
**Results:** A total of 95 women were evaluated, with 80 (84.2%) had type I of endometrium and 15 (15.8%) had type II of endometrium. The mean age of the patients was 35.42 years in both groups. The ovarian stimulation was performed with gonadotropin-releasing hormone antagonists in 90 patients (94.7%). The type I of endometrium was found in 93.75% of the women of the patients who used the antagonist protocol. All the patients showed endometrial type II were submitted to the stimulation with antagonist. The rates of total pregnancy (43.75%, 60.0%), clinical (40%, 53.3%), and pregnancy in progress (84.3%, 100%) respectively, for types I and II showed similar proportions. Patients with type I of endometrium had a higher number of abortions (15.63% versus 0.00%), while patients with type II of endometrium had a higher proportion of patients with a biochemical pregnancy (11.11% versus 8.57%).
**Conclusion:** The classification of the endometrium in the ultrasonographic aspect varies in the cycles of induction and interferes in the implantation rates, as well as the thickness. Through this descriptive study it was possible to demonstrate that the vast majority of patients presented type I endometrium (84.2%), after induction of ovulation, to perform ICSI. However, it was not possible to identify difference in the success rates of the technique between the groups because the vast majority of the patients presented type I endometrium.

### P-20. IVF/ICSI. Pregnancy chance by morphologic classification and number of D4 transferred morulae

**J.P. Rocha[1], M.P. Batista[1], G.C. Borges[1], E.C. de Castro[1], C.L.A. de Castro[1], V.A. de Oliveira[1], R.S. Florencio[1]**

*[1]Humana Medicina Reprodutiva - Goiânia/GO.*

**Objective:** To determine the chances of chemical ,clinical and ongoing pregnancies by D4 transfer, and the kind of morula (Humana 3 transfer classification).
**Methods:** Two hundred and sixty-three transfers on day 4 after collect (D4), from 2015 to 2017, including in vitro fertilization with their own eggs, up to 39 years of age. Out of these 05 with <7mm endometrium size, 12 cycles of moderate or difficult transfer and that cases where not possible to evaluate by pictures (65 cycles). Therefore, the remaining transferring to be studied were a hundred eighty-one.
The morulae were classified as: Type 4 morula(M4), when ≥80% compacting; type 3 morula(M3), when 50-79% compacting; type 2 morula(M2), when <50 compacting of the blastomeres and type 1 morula(M1), when ≥12 blastomeres with no compacting. On the transferring we used a transfer classification (Humana 3, 2017), which consisted of, for D4: type 1 (2 M4 morulae), type 2 (1 M4 morula or 2 M3 morulae), type 3 (any number of embryos embryos with ≥6 blastomeres up to M2 morulae), type 4 (any number of embryos ≤6 blastomeres).
The morulae were shot and classified within 92 to 96 hours after injection and reanalysed through the pictures.
**Results:** The average age for transferring was 33.44 years on D4. The percentage of transfer on D4 with regards to the presence of, at least, 2 D3 embryos, was 100%. Out of the transferring total, 63/181 (34%) were type 1 transferring; 47/181(25.96%) type 2, 50/181(27.62%) type 3, 21/181(11.60%) type 4. The chemical, clinical and ongoing pregnancy rates were, at D4 total 36.40%, 30.3%, 18.7% respectively, and by transferring type: type 1(55.50%; 47.60%; 30.1%), type 2( 36.10%; 27.60%; 12.70%), type 3 (18.00%; 16.00%; 14.00% ), type 4(23.8%; 9.52%; 0%). Clinical pregnancy rate type 1/ type 2 ($p$=0.0043), type 2/3 ($p$=0.1301), type 3/type 4 ($p$=0.7509).
**Conclusion:** The different types of transferring, showed difference on the pregnancy chance, when we analyzed the morulae characteristics, regarding the compacting degree, regardless of any other embryonic variables. The T1/T2 clinical and ongoing pregnancy difference were statistically significant in this sampling.

### P-21. IVF/ICSI. Chemical, clinical, and ongoing pregnancy rate of top embryos transferring D4 versus D5-6

**R.S. Florencio[1], M.N.C. Rocha[1], J.P. Rocha[1], G.C. Borges[1], M.R. Borges[1], M.C.C.F. Finotti[1], F.C. Santos[1]**

*[1]Humana Medicina Reprodutiva - Goiânia/GO.*

**Objective:** To compare the chemical, clinical and ongoing pregnancy rate from type 1 transferring (top embryos) of D4 versus D5-6 and other types of transfer.
**Methods:** A hundred eighty-one D4 transfer (2015-17) of ≥2 (2 PN) patients were compared to the D5-6 ,79 transfer (2016) for ≥6 (2PN), where we evaluated the pregnancy chance for the two groups on type1 (D4)= 2 morulae ≥80% compacting and D5= 2 blastocytes ≥3BB). IVF/ICSI results were analyzed with their own eggs up to 39 years of age. In the D4 group, type1(63 patients) and D5-6 group, type 1(56 patients), we assessed the pregnancy chance and in the type 2 transferring (1 morula ≥80% compacting or 2 morulae 50-79% compacting), type 3 (≥6 blastomere embryos up to morulae <50% compacting) of D4 (97 patients) and in D5 group (19 patients).
**Results:** The average age was similar between the two group 31.80/31.40 years. The pregnancies rate, in type1 transferring for the groups were: chemical 55.57%(D4)/62.50%(D5) $p$=0.2611; clinical 47.60%(D4)/50%(D5) $p$=0.8552, and "ongoing" 30.10% (D4)/ 32.14%(D5) $p$=0.8458. Ongoing pregnancy rate in type 2 and 3 were 13.40% (D4)and 10.52% (D5), $p$=0.9894.
**Conclusion:** In this D4/D5 transferring sampling comparison there was no difference of any pregnancy rate between the two groups.

### P-22. Live birth after delayed intracytoplasmic sperm injection of immature oocyte- a case report

**D.P.A.F. Braga[1,2], R.C. Ferreira[1], C.C.V. Brogilato[2], B.F. Zanetti[2], A.S. Setti[1,2], A. Iaconelli Jr.[1,2], E. Borges Jr.[1,2], C.A.R. Iaconelli[1]**

**Case report:** A 35-year-old female patient with diagnosis of primary infertility due to peritoneal factor, with endometriosis and tubal obstruction due to bilateral salpingectomy. Complete seminal analysis showed normal sperm concentration and motility, and morphology of 2%, characterizing teratozoospermia. The couple was referred to the IVF center and intracytoplasmic sperm injection (ICSI) was indicated. Two cycles of ovarian stimulation (COS) were performed. The first COS was achieved using a total dose of 2550 IU of recombinant (rFSH) and urinary (uFSH) FSH, pituitary suppression was performed using GnRH antagonist (antGnRH) and final oocyte maturation was triggered using with recombinant hCG (rhCG). Fourteen follicles were punctured and 9 oocytes were obtained, in which 3 were mature and 4 were maintained in culture until next day for in vitro maturation, totalizing 7 vitrified oocytes for accumulation. The second COS was performed with rFSH and uFSH (total dose of 2325 IU), pituitary suppression with antGnRH and trigger with rhCG; 15 follicles were punctured and 10 oocytes were obtained, of these 8 mature oocytes and 1 with in vitro maturation. ICSI was performed with 9 fresh oocytes plus 6 heated oocytes from the previous cycle. Fertilization was checked 16 hours post-injection. After 16h of ICSI, all oocytes were found to have normal fertilization. No transfer of fresh embryos was performed due to inadequate endometrium, and 8 embryos were frozen in day +3. Two embryo transfer cycles were performed with 17-β-estradiol endometrial preparation in the first phase of the cycle, and micronized progesterone was added in the second phase. In the first one, 8 embryos were thawed in day +3 and, 1 expanded blastocyst and 1 expanded blastocyst with hatching were transferred in day +5, without pregnancy success. Two embryos were refrozen. In the second thawing cycle, refrozen embryos were heated and 1 expanded blastocyst and 1 embryo at the beginning of cavitation were transfered, resulting in biochemical gestation. After 7 months of the second ovum pick-up, Third COS was started. The antral follicles were showed the presence of only 3 follicles, demonstrating a great loss of ovarian reserve in this period. A total dose of 1650 IU of rFSH and uFSH was administered. Pituitary suppression was performed with antGnRH and trigger with rhCG. The estradiol dose on the day of hCG administration was 584 pg/mL. On the day of ovum pick-up, three follicles were punctured and only one oocyte was retrieved. After denudation, it was observed that the oocyte was at germinal vesicle stage, thus it remained in culture until the next day. On the morning of day +1 of the cycle, the oocyte had reached metaphase II stage and was injected with ejaculated spermatozoa. Fertilization was checked 16 h post-injection and the presence of 2 pronuclei was observed. On the second day of embryonic development, the third day of the cycle, the embryo had 4 similar cells without fragmentation, and the next day 8 similar cells without fragmentation, showing good embryonic quality. The embryo remained in extended culture until day 5 of the embryo, day +6 of the cycle, and was transferred as expanded hatching blastocyst. Luteal phase support was performed with micronized progesterone from the day after the puncture and maintained for up to 12 weeks. After ten days, a positive serum βhCG result was obtained. At 6 weeks gestation, one gestational sac with fetal heart beat was observed by ultrasonography. The pregnancy progressed well and a newborn with 49cm and 3450kg was delivered by cesarean section, at 38 weeks' gestation, without complications.
**Comments:** In patients with poor ovarian response, ICSI performed in oocytes matured in vitro, even the day after follicular puncture, may be a viable alternative, with positive results, even if rare.

### P-23. Identified microorganisms on clean room surface samples from an Assisted Reproduction laboratory

**K.Q. Poletto[1], Y.A.R. de Lima[1], D.F. Rezende[1], E.C. de Castro[1], M.S. Approbato[1]**

**Objective:** To identify microorganisms in surface samples from a clean room in an assisted reproduction laboratory.
**Methods:** Surface samples from the grids of two embryo incubators (incubator grid-1 and -2) and from the laboratory bench top were collected monthly for three months. RODAC® contact plates were gently pressed in these locations for 10 seconds. The plates were incubated at 37 °C for 48 hours, sealed and incubated for another 48 hours in order to perform the Colony Forming Units (CFU) counting. Microorganisms were identified through biochemical and enzymatic tests.
**Results:** A non significant variation in the CFU counting was observed during the sampling time ($p$=0.0635). Regarding the bench top surface, in the first time point 60 CFUs were identified, 23 CFUs were counted in the second month and 32 CFUs at the third time point (mean=38.3; SD=±19.3). Concerning the incubator grid-1, a total of three CFUs was identified at the first month, no CFUs were counted in the second collection and one CFU was identified at the third month (mean=1.3; SD=±1.5). Regarding the incubator grid-2, five CFUs were identified at the first month, no CFU at the second one and one CFU at the third one (mean=2.00; SD=±2.6). The following microorganisms were isolated: Enterococcus sp, Staphylococcus saprophyticus, Staphylococcus epidermides, Gram bacilli positive and hyaline septate filamentous fungi.
**Conclusion:** Microbial counts were in accordance with ISO 5, 7 and 8 standardization required for IVF procedures. An environmental microbiology monitoring program is required for IVF laboratories in order to avoid contamination. Standardizing some parameters such as an efficient sanitization and the optimal frequency of air filtration system exchange may significantly reduce the number of microorganisms in the laboratory environment.

### P-24. Otimizing medical consultation time in Assisted Reproduction

**A.M. D'Avila[1], L.C. Bianchi[1]**

*[1]Embrios centro de Reprodução Humana.*

**Objective:** The role of nursing in the field of human reproduction has been growing continuously, especially with regards to patient care and humanized care. Patients with infertility carry expectations, among them, of finding a trustworthy and welcoming environment. The nurse serves as a link between the doctor and patient, conveying safety, peace-of-mind and personalized assistance. Objective: To evaluate the impact of applying a questionnaire and the nursing care precedent to the medical consultation about the time of the latter and about the waiting time to perform a consultation when scheduled by the patient.
**Methods:** After scheduling an appointment through the telephone, by social media or in person, a questionnaire containing 40 questions for the woman and 37 questions for the man was sent by e-mail and should be returned until one day prior to the consultation. The nurse sees the patient/couple for 30 minutes before the medical consultation, when general issues and patient/couple expectations are discussed before the doctors sees them.
**Results:** There was a mean reduction of 90 to 60 minutes in the consultation with the physician, increasing the average number of consultations from 2.5 to 3.5 consultations/shift. The waiting time for the consultation after the appointment was reduced from 90/100 days to 50/70 days.
**Conclusion:** The questionnaire and the nursing care prior to the medical consultation optimized the time of care because it could focus on the clinical aspects, enabling more appointments and consequently a decrease in waiting for care.

### P-25. Ovarian vitrification imply in gene expression changes related with ultrastructural adaptations that maintain the ovarian and oocyte viability

**L.A.A.C. Pereira[1], T.M. Segatelli[2], E.C. Jorge[2], P.H.A. Campos-Junior[1]**

**Objective:** In the last decade, the rate of survival of cancer patients has significantly increased with the use of chemo and radiotherapies. However, these treatments are gonad-toxic, as they induce the apoptosis of non-growing and growing follicles. The result is an increasing number of patients with premature ovarian failure and a considerable number of infertile young women. Ovarian tissue vitrification is a promising oncofertility technique, and can be used to preserve the entire follicular pool, being able to restore the patient's fertility and also its endocrine function. In a recent work, we could successfully establish a new protocol for ovarian tissue vitrification followed by autotransplantation, showing a complete recovery of fertility and production of functional oocytes in mice. In this work, we aimed to evaluate the molecular and cellular effects of the murine ovarian tissue vitrification.
**Methods:** The molecular effects were evaluated by RNAseq. Fresh (n=3) and vitrificated (n=3) ovarian tissue samples were obtained 20 days after transplantation. Total RNA was isolated using Trizol and RNA libraries and sequencing were performed using Illumina protocols. FASTQC was used to measure the quality of the raw data and the mapping was performed by STAR. Reads were quantified by HTseq, differential expression analysis were performed using Deseq2 ($p<0.05$), and the most represented functional classes were analyzed by DAVID, using the "Cellular Component" term of gene ontology (GO-CC). The up and down-regulated genes were validated using qRT-PCR. For this, 0.5µg of total RNA was converted in cDNA, using random hexamers. The relative quantification of gene expression was quantified using specific primers on an ABI 7500 SDS instrument, using Gapdh as the reference gene. Vitrification cellular effects were investigated using Transmission Electron Microscopy (TEM) in the ovarian tissues fixed and processed in routine protocol.
**Results:** In the present study, 332 genes were found as up-regulated and 291 as down-regulated in vitrified tissue, compared to the control. On GO-CC, the top 5 up-regulated genes were found to be related to cytoskeleton, cell surface and cell projections. On the other hand, the top 5 down-regulated genes were related to extracellular space, endoplasmic reticulum (ER), Golgi apparatus and integral component of plasma membrane. qRT-PCR results could validate the RNAseq data. TEM showed an abundance of both cytoskeleton and junctions components in the vitrified samples, compared to the control. Otherwise, organelles such as endoplasmic reticulum, mitochondria and polyribosomes (free or associated to the ER) were less frequently observed in vitrified samples, compared to the control.
**Conclusion:** Taking together, these molecular and structural alterations may have occurred as an adaptive response of ovarian cells to the vitrification process, demonstrating the success of this technique due to oogenesis recovery.
**Acknowledgment:** Fapemig, CNPq, CAPES.

### P-26. Seminal analysis in patients with normal and altered BMI from an Assisted Reproduction clinic of Curitiba, Paraná

**D. Scaraboto[1], R.Y. Cho[1], I.T. Saruhashi[1], L.J.R. Centa[1]**

**Objective:** To evaluate the main factors of the seminal analysis (volume, concentration, motility and morphology) and its relationship with BMI (Body Mass Index) of 330 patients who sought ANDROLAB - Human Reproduction and Andrology Medical Center and Laboratory in Curitiba for sperm testing.
**Methods:** Data collection was performed through a questionnaire applied to patients who sought the private laboratory to perform semen analysis. The BMI was calculated from the data provided in the questionnaire with the formula IMC = weight (kg)/height (m)•, being considered with adequate weight the patients with BMI greater or equal to 18.5 and less than 25; overweight those with BMI greater than or equal to 25 and less than 30 and obese with BMI greater than or equal to 30. This classification follows the recommendation of the Brazilian Ministry of Health. To facilitate the visualization of the results, the patients were separated into two groups: those with a BMI greater than 25, referred to as "high BMI" and with a BMI below 24.9 "adequate BMI". The seminal analysis was performed according to WHO standards in its guide *Laboratory Manual for the examination and processing of human semen of 2010*. Values considered normal were: seminal volume ≥1.5 mL, concentration ≥15 million spermatozoa per mL, motility ≥32% progressive moving spermatozoid and morphology at least 4% normal spermatozoid (strict Kruger standard).
**Results:** The mean age of the patients was 34 years (14-64). The mean results were: volume 3.8mL (0.5mL-12.5mL), concentration 64.7 million spermatozoa per mL (0 - 436), motility 37% progressive spermatozoa (0% - 85%) and morphology 4% of normal spermatozoa (0% - 9%). In general, 5% of the patients presented volume reduction, 19% decrease in quantity, 35% worsening of motility and 38% morphological alterations. Regarding weight, 65% of the patients had a high BMI. 1% of the patients had BMI lower than 18.5, being considered undernourished and, therefore, were excluded from the groups analyzed. In the group "adequate BMI" the incidence of changes were: 1% of changes in volume; 13% of quantity; 31% motility and 25% morphology. Whereas in the group "BMI High" the changes were: 7% of volume; 26% of quantity; 36% motility and 40% morphology. There was a statistical difference in volume ($p$=0.012) and concentration ($p$=0.078). Motility ($p$=0.400) and morphology ($p$=0.79) did not show a statistically significant change.
**Conclusion:** According to data from IBGE, the population of overweight and obese people has increased worldwide. Overweight in adults in southern Brazil is around 55%. As the rate of patients with high BMI at the center where the study was applied was higher than the Brazilian rate, there may be an indication of trend in the direct relationship between obesity/overweight and infertility. The parameters semen volume and concentration changed according to the BMI difference in a statistically significant way ($p<0.05$). The other parameters did not present statistical difference.

### P-27. Semen analysis patterns in patients from an Assisted Reproduction clinic in Curitiba, Paraná

**D. Scaraboto[1], I.T. Saruhashi[1], R.Y. Cho[1], L.J.R. Centa[1]**

**Objective:** To evaluate the main characteristics of the seminal analysis (concentration, motility and morphology) from 383 patients who sought the ANDROLAB - Human Reproduction and Andrology Medical Center and Laboratory in Curitiba for sperm examination.
**Methods:** Data collection was performed through a questionnaire applied to patients who sought the private laboratory to perform sperm from November 2017 to May 2018 and signed the authorization to use their data in studies. Seminal analysis was done following the WHO standards in its guide. Laboratory Manual for the examination and processing of human semen of 2010. Vasectomized patients were excluded. Values considered normal were: seminal volume ≥1.5mL, concentration ≥15million spermatozoid per mL, motility ≥32% progressive mobile spermatozoid, and morphology at least 4% normal spermatozoid (Kruger's strict standard).
**Results:** The mean age of the patients was 35 years (14-70). Seminal parameters of volume, concentration, motility and morphology were evaluated. The mean results were: volume 3.8 mL (0.5mL-12.5mL), concentration 64.4 million spermatozoa per mL (0-436), motility 36% progressive spermatozoa (0% - 85%) and morphology 4 % of normal spermatozoa (0%-9%). Overall, 5% of the patients had a change in volume, 19% had a change in quantity, a 34% change in motility and a 39% change in morphology.
**Conclusion:** Recently, UNICAMP published a study with almost 18,902 patients, showing concentrations of 48 million spermatozoa per mL, progressive motility of 36% and morphology of 3.7% normal. These values are in agreement with those found in the evaluated center. It is worth bearing in mind that patients who seek a sperm exam at a clinic specializing in infertility commonly have a search indication for infertility research.

### P-28. Patients' profile of social egg freezing in a Private Center in São Paulo, Brazil: evaluation of 5 years of experience

**M. Cavagnoli[1], C. Gomes[1], A.R. Lorenzon[1], P.C. Serafini[1,2], E.L.A. Motta[1,3]**

**Objective:** Over the past decades, the average age of women's first childbirth has increased. The cause for delaying motherhood is multifactorial, including social and economic reasons. Because of this, age-related infertility has increased. From 2013, oocyte cryopreservation stopped being an experimental technique and started to be considered efficient for non-medical indication for fertility preservation. Our goal is to determine the profile of patients who underwent social egg freezing in a private center in São Paulo, Brazil.
**Methods:** This is a cross-sectional study from 381 patients (496 cycles), which underwent social egg freezing from Jan 2013 to December 2017, at Huntington Medicina Reprodutiva, São Paulo, Brazil.
**Results:** Average age of women who went social egg freezing decrease from 2013, n=59, to 2014, n=80 (37.8 vs 36.7 years old $p$=0.0312), and then has not changed through years (36.7, 36.4, 36.6 and 36.7 from 2014 to 2017, $p$=0.9237, 2015, n=97, 2016, n=123, 2017, n=137 patients). The main reason for egg freezing is fertility preservation (89.83%, 86.25%, 93.81%, 79.67% and 73%, 340 patients, 411 cycles, 2013-2017, average age is the same to general social egg freezing), followed by low ovarian reserve (1.69%, 6.25%, 1.03%, 5.69% and 17.52%, 32 patients, 41 cycles, 2013-2017), which has increased in last year (17 patients, 24 cycles, 2017). Considering only patients from fertility preservation, oocytes retrieved number is higher in patients under 35 years old (97 patients, 113 cycles) when compared to patients after 36 years old, (238 patients, 298 cycles (12.96±8.64 vs 9.70±6.79, $p$<0.0001), and MII/number of vitrified oocytes as well (8.89±6.26 vs 7.13±5,76, $p$=0.004). The percentage of MII oocytes (MII/OPU) has not changed in these groups (66.76% vs 70.94%, $p$=0.0939).
**Conclusion:** Although there is an increase in the number of patients seeking for social egg preservation, the average age of women that underwent social egg freezing for fertility preservation is still higher for this purpose. Since there is an increase in number of oocytes vitrified in patients under 35 years old compared to patients after 36 years old and considering women under 35 years old has better quality oocytes, fertility preservation should be counselling to patients under 35 years old to increase number of vitrified oocytes and chances to provide better reproductive outcomes.

### P-29. Cytogenetic profile of the infertile couples evaluated by human reproduction genetics at the Hospital Materno Infantil de Brasilia

**C.T.N. Medina[1], B.F. dos Reis[1], A.A. Crispin[1], G.P.M. Antonialli[1], A.C.R. Leite[1], B.R. Versiani[1], E.C.N. da Silva[1], S.M. do Couto[1], G.R. Vasconcelos[1], T.M. de Oliveira[1], M.T.O. Cardoso[1], R.R. Costa[1], E. Pimental[1], N. Zavattiero[1]**

**Objective:** Infertility is defined as the inability to conceive naturally after one year of regular unprotected sexual relations. This condition affects around 15% of all reproductive age couples. The rate of structural and numerical chromosomal alterations observed in infertile individuals is known to be higher than in the general population, and is one of the most important causes of this disorder, both in men and in women. The main objective of this study was to determine the frequency of chromosomal alterations observed in the karyotypes of infertile couples evaluated by the Medical Genetics Service of the Maternal-Infantile Hospital of Brasilia.
**Methods:** Case files were reviewed for 278 couples sent for genetic evaluation between 2008 and 2017. Each couple was classified according to history and semen analysis, and karyotype analysis was solicited according to each case. Karyotyping was performed on peripheral blood lymphocytes by using the Giemsa trypsin banding (GTG) technique. For each sample, at least 20 cells were analyzed, and in cases of mosaicism, the count was expanded to 30-50 cells, as necessary. Only alterations found in two or more cells were considered for mosaicism. Polymorphisms were not considered as alterations.
**Results:** The average age among women was 32.16 years, and for men was 36.12. Among the main causes of infertility, male issues accounting for 42.08% of cases, while female causes were detected in only 5.39% of cases. In 30.57% of cases the couples were referred due to a history of recurrent miscarriage, in 15.1% of cases for genetic counselling, consanguinity, previous occurrence of fetal malformation, or parental genetic disease, and 6.11% of cases the infertility was due to male and female issues. In 0,71% of cases it was not possible to identify the cause. We detected 18 cytogenetic alterations (5.29%). Among men, 6.28% karyotypes were altered and among women, 3.24%. In agreement with data in the literature, men with karyotype alterations presented azoospermia or severe oligozoospermia. For the women, the majority of cytogenetic alterations encountered were observed in women with risk factors such as recurrent miscarriage or children with malformations, except for one woman referred for idiopathic conjugal infertility, whose karyotype revealed a balanced translocation.
**Conclusion:** The current work evaluated the prevalence of chromosomal abnormalities in infertile couples referred to an outpatient clinic specializing in reproductive genetics. There is no consensus in the literature on the indication of karyotype in infertile couples. Although our data are in line with other studies, indicating male karyotyping in azoospermic men or with severe oligozoospermia, it is difficult to interpret the data in relation to the female sex. Cytogenetic screening has been suggested only in women who present with primary amenorrhea, premature ovarian aging and several recurrent pregnancy losses. In this study, however, a balanced translocation was detected in a woman without factors suggestive of cytogenetic alteration, except for conjugal infertility. There is a general consensus that the incidence of abnormal chromosome analysis is increased in couples who are candidates for ART. Detecting such changes allows for adequate genetic counseling, informing couples about early abortion or transmission of genetic anomalies to the conceptus.

### P-30. Preservation of fertility in women submitted to gonadotoxic treatments in the public health system of Minas Gerais

**J.K. Rodrigues[1,2], C.S. Monteiro[1], T.E. Silva[1], C.M. Canabrava[1], I.K.D.C. Cruzeiro[1,3], F.A.N. Pereira[1,3], F.M. Reis[1]**

**Objective:** To discuss the difficulties and importance of a fertility preservation program in the public health system directed to patients who will undergo gonadotoxic treatments.
**Methods:** Data collection was performed from records of the Assisted Human Reproduction Sector and database of the Oncology Sector of the Hospital das Clínicas, Federal University of Minas Gerais from 2013 to 2017. The number of patients treated in the Oncology sector was estimated based on the number of authorizations for treatment approved month to month as an approximate number of patients treated. Patient confidentiality was preserved.
**Results:** During the study period, we observed that 8846 patients were treated, being 6171 female, and of these, 446 (7%) were on reproductive age. Among these women who underwent chemotherapy, 16 patients preserved their gametes because of the potential risk of developing ovarian failure. This number corresponds to 4% of women of reproductive age who underwent gonadotoxic treatment in the same institution. Among the diagnosed diseases were breast cancer, non-Hodgkin's lymphoma, chronic myeloid leukemia, cervical neurofibrosarcoma, Castleman's disease, cervical/mediastinal dysgerminoma and sickle cell anemia.
The preservation of fertility in patients undergoing gonadotoxic treatment is in constant development and enables the preservation of the reproductive health of patients in childbearing age who are subject to infertility. It is imperative that these patients be advised of the option of fertility preservation techniques, both pre- and posttreatment, so that in the future the woman will have the option of becoming pregnant with her own eggs. In the US approximately 40% of the patients are referred for fertility preservation. On the other hand, in Brazil, despite the precariousness of the Brazilian database on this subject, it is believed that the fertility preservation numbers are smaller. Due to the lack of orientation of the patients by the doctors, in the Unified Health System (SUS), the situation of patients who need to preserve their fertility becomes more laborious, in addition to bureaucratic processes, the government does not have a specific program that prioritizes effectively and urgently these patients. Based on this context, our study verified that in the last two years, the number of female patients who had undergone preservation of fertility in the Oncology and Human Reproduction Sector of the Hospital das Clínicas, Federal University of Minas Gerais, doubled to the sum of years from 2013 to 2015. However, the number of patients who performed this procedure is still low. Regarding the orientation of these patients regarding the option of preserving reproductive health pre-treatment gonadotoxin, only one did not have its fertility preserved before the chemotherapy.
**Conclusion:** Despite the increase in the number of patients who have undergone preservation of fertility in the last two years, the total number of patients is still low, compared to the number of women of childbearing age who passed the Oncology Sector of the Hospital das Clínicas, Federal University of Minas Gerais and are undergoing some gonadotoxic treatment. Therefore, our study evidenced the importance of orienting this group of patients on the techniques of preservation of fertility available in SUS and the need for a specific flow for this sector, in order to expedite the approach of patients at risk of ovarian failure due to treatments gonadotóxicos.

### P-31. Prevalence of subclinical hypothyroidism in infertile patients

**D.F. Rezende[1], Y.A.R. de Lima[1], K.Q. Poletto[1], M.S. Approbato[1], E.C. de Castro[1], A.V.S. Moraes[1]**

**Objective:** Determine the prevalence of subclinical hypothyroidism in infertile patients of a public service of human reproduction.
**Methods:** This is a cross-sectional retrospective study, with a quantitative approach of data from patients attended at the Human Reproduction Laboratory of the Clinical Hospital from the the Federal University of Goiás, from August 2002 to March 2018, and the information was taken from the database Sisfert 2.0. Serum thyroid-stimulating hormone (TSH) was evaluated as the reference value for diagnosis of subclinical hypothyroidism in infertile patients, TSH values above 2.5 mIU/L. For analysis of the variable, a significance level of 5% ($p$<0.05) was used. All the patients attended in the service during the specified period, aged between 21 and 40 years, who were in the database of STH serum values, were included in the study.
**Results:** The initial n sample was 1784 patients and the final n sample was 1554. Two hundred and thirty patients were excluded from the study because they were older than 40 years or presented incomplete data in the database. The prevalence of subclinical hypothyroidism was 20.07% (n = 312, 95% CI, 18-22%). The mean age in the group studied was 34.3 years (SD±4.2).
**Conclusion:** The incidence of subclinical hypothyroidism in the reproductive age population is approximately 6%. In infertile patients, using TSH values above 2.5 mIU/L for the diagnosis of subclinical hypothyroidism, the prevalence of this disease in this group is increased. Although there is no consensus in the scientific literature regarding the limiting value of this hormone for infertile patients, the most frequently used value for early pregnancy diagnosis is used in an attempt to minimize the potential risks associated with hypothyroidism during pregnancy. The diagnosis of this condition is important in women assisted in assisted reproduction services, since the treatment of subclinical hypothyroidism may be associated with an improvement in the rates of clinical gestation and live births and a reduction in the rate of abortion.

### P-32. Nursing care protocol to the patient submitted to Assisted Reproductive Technique - Safe Surgery

**E.A.V. Righetti[1], A.F. Gonçalves[1], S.F.F. Magrin[1], M.P.da Luz[1], S.S. Resende[2], V.G. Maziero[1]**

**Objective:** Elaborate a nursing care protocol and develop a work tool to be applied by the multidisciplinary team during the surgical activities.
**Methods:** The bibliographic survey method was used. The research was elaborated based on 26 scientific articles indexed in magazines, manuals and books of nursing and of the health area published between the years of 1986 to 2018, in Portuguese, Italian, French and English.
**Results:** Assisted reproduction/in vitro fertilization is indicated when the couple has a fertility problem, either by tubal factors, when the tubas are obstructed; or by seminal changes, such as, low number of sperm motility changes, among others. After the medical consultation and the definition of the procedure to be performed, it is up to the nursing to guide the couple and resolve their doubts about the indicated treatment. It is necessary that the team is able to clarify in an objective way about the importance of the examinations and the correct use of each medication, schedules and route of administration. After evaluation of procedures performed in assisted reproduction, it was observed the need to draw up the checklist and the involvement of all the staff in this protocol. The definition of the term "patient safety" is the reduction, to a minimum acceptable, of the risk of necessary damage associated with health care. The tool used in this environment to check the conditions that guide a safe surgery is the checklist or safety checklist of the surgical procedure, also known as Time Out, where it will follow the principles of simplicity, wide applicability and measurement possibility reduces the occurrence of incidents and adverse effects.
**Conclusion:** The analysis of the data allowed the development of a checklist for safe surgery. The elaboration of a nursing care protocol shows the new techniques used as well as the ethical vision of the procedures to acquire critical opinion of the situation and interact with the patient always involved with an accessible language. The purpose of this protocol is to determine the measures to be taken to reduce the occurrence of incidents, adverse events and surgical mortality, making it possible to increase the safety in the relation of procedures.

### P-33. Case Report: Recovery of testicular function after prolactinoma treatment

**J.F. de Macedo, M.R. Oliveira, L.M.O. Gomes, G.C. de Macedo, G.C. de Macedo, D.O. Gomes, L.C. Maciel**

**Case report:** Study describing clinical case of prolactinoma followed by hypogonadotrophic hypogonadism, its evolution and treatment. This is a 36-year-old male patient who looked for medical care in January 2012, referring to episodes of holocranial headache, decreased libido and erectile dysfunction. Laboratory diagnostic investigation was done, revealing normal testosterone, high prolactin and normal sperm levels. The magnetic resonance imaging of the skull confirmed extrafascial expansive lesion and supra seal. Treatment with Cabergoline 0.5 mg was instituted with improvement of clinical symptoms. After a three-year treatment, the patient underwent further tests resulting in normal follicle stimulating hormone (FSH) and luteinizing (LH) and decreased testosterone. There was a prolactin normalization, confirmed by magnetic resonance imaging, which showed normalization of pituitary images, but there was a gradual decrease in FSH, LH and testosterone. Azoospermia was confirmed straight after a spermogram test done in July, 2016. In view of the hypogonadotrophic hypogonadism, a treatment with Clomiphene Citrate was performed with progressive normalization of FSH, LH and decreased testosterone, though a new spermogram revealed a small concentration of spermatozoa with progressive motility. The treatment was replaced by tamoxifen citrate and human chorionic gonadotrophin 5000 IU. In seven months of treatment the patient recovered the seminal parameters, but with decreased testosterone. In this context, the patient's semen was frozen in order to preserve its fertility. The treatment with Cabergoline was kept, suspending the other medications and testosterone cypionate was instituted due to the loss of libido complaint and erectile function, thus, a new azoospermia was confirmed in January 2018.
**Comments:** Cabergoline administration is argued to be the gold standard treatment of prolactinomas. It is verified that the impotence and decreased libido are clinical manifestations that occur in patients who suffer from hyperprolactinemia, even in those with normal testosterone, but in patients suffering from decreased hormone, they need administration of exogenous androgens and gonadotrophins. In this case, the patient presented alterations in hormone levels and fertility during the use of cabergoline, so that clomiphene citrate treatment was started, whose use restores the production of endogenous testosterone, since the drug acts on the hypothalamic-pituitary axis, increasing the production of FSH and LH. Tamoxifen acts similarly and both constitute treatment methods with effective responses in improving seminal parameters. However, treatment with tamoxifen may lead to side effects such as cardiovascular disorders, development of obesity, hepatic steatosis, and gastrointestinal irritation. HCG therapy is coadjuvant in treatment for replacing the action of LH, promoting the restoration of testosterone. In this study, the use of tamoxifen citrate and hCG led the patient to recover the seminal parameters, but testosterone remained reduced. After the freezing and the introduction of exogenous testosterone, a new azoospermia picture was confirmed, evidencing the consequences of the use of exogenous testosterone that results in the negative feedback of the hypothalamus, decreasing secretion of the gonadotrophic hormones and inhibition of spermatogenesis. In view of the complexity and variety of results in the treatment of hypogonadodrophic hypogonadism with hormone replacement, it is important to highlight the importance of freezing the patient's semen in order to preserve their fertility, sexual and emotional quality of life, since he is a young individual with future paternity.

### P-34. Assisted Human Reproduction under the public healthcare system: implementation of comprehensive care policies and the current scenario

**R.S. Garcia, R.A. de Moraes, M.A. Turci**

**Objective:** The purpose of the study is to analyze the governmental policies regarding infertility treatment. Furthermore, promote a revision of the current situation of places that perform procedures of assisted human reproduction in patients coming from the Brazilian Public Health Service (SUS). The purpose is based on the heterogenic public that want this kind of procedure, although they do not have access to it, because of the high prices of the procedures that involve assisted human reproduction.
**Methods:** The study is based on quantitative and qualitative research. The qualitative research features documental revision about the public policies available for infertility treatment, as well as the institutions that receive support from SUS. The quantitative research features data analysis published by The Brazilian Health Ministry and the National Embryo Production System/Anvisa.
**Results:** The study has identified three policies that already exist to implement assisted human reproduction procedures in the public health system. The first one is the National Policy of Integral Attention to Women's Health launched in 2004, that aims to amplify and qualify the attention given to family planning, including infertility assistance. The second is the National Policy on Comprehensive Care in Assisted Human Reproduction that established the support for infertility treatment in the public health system in 2005. The third policy is the ordinance number 3,149 that determined an investment of ten million reais to nine institutions that provided assisted human reproduction in the public health system in 2012. However, despite the investment in 2012, resources available are insufficient for the high demand of infertile couples that seek reproduction procedures. The Clinical Hospital of The Federal University of Minas Gerais is an example of the Brazilian reality. In 2006, this hospital had 2,100 couples in the queue that waited from three to four years for a consultation. Another example is the Clinical Hospital of Ribeirão Preto and the Fêmina Hospital in Porto Alegre, where couples need to wait for about two years for a consultation. Although they are hospitals that provide assisted human reproduction with public resources, patients have to pay for medicines that can cost more than five thousand reais. The Maternal Infant Hospital in Brasília is an exception, where the treatment is totally free, including all medication. As a quantitative analysis, the research showed that there were 78,216 frozen embryos in Brazil in 2017 after analysis of 146 assisted human reproduction clinics. The increase of demand for the procedures as well as the increasing number of clinics and hospitals that perform them, became even more evident comparing the numbers of 2017 with 2007. In 2007, 32,181 embryos were frozen in 50 clinics analyzed.
**Conclusion:** The SUS principle of comprehensive care is unfulfilled because of all challenges regarding fertilization procedures. Assisted human reproduction policies need to be implemented more effectively. Furthermore, more financial support is necessary so that in the future a larger proportion of the population with infertility problems can benefit from the technology available in assisted reproduction. This way, the healthcare system in Brazil can be truly comprehensive.

### P-35. Pregnancy rates between natural and artificial cycles of women submitted to frozen embryo transfer: a meta-analysis

**K.Q. Poletto[1], M.P. Lobo[1], M. Giovanucci[1], Y.A.R. de Lima[1], M.S. Approbato[1], E.C. de Castro[1]**

**Objective:** To compare pregnancy rate among natural and artificial cycles of women submitted to transfer of frozen embryos.
**Methods:** A systematic review was performed by PubMed search using the following algorithm: (endometrial[All Fields] AND preparation[All Fields]) AND (("freezing"[MeSH Terms] OR "freezing"[All Fields] OR "frozen"[All Fields]) AND thawed[All Fields]) and (natural cycles) AND (artificial cycle). Inclusion criteria: prospective and retrospective cohort studies. Exclusion criteria: use of hCG in the natural cycle, oocyte donors, and use of disused freezing techniques. Data were analyzed with SPSS v. 23 software and with a significance level of 5%. The meta-analysis was performed with RevMan 5.3 software. $I^2$ was calculated.

**Results:** A total of 709 articles was retrieved. Five studies fulfilled the inclusion and exclusion criteria. Among these studies, a total of 8,968 natural or artificial cycles was analyzed. A contingency table compared the results of the natural and artificial cycles and the number of clinical pregnancies obtained in each selected article. $I^2$ test resulted in high statistical heterogeneity ($I^2$=77%). Studies by Morozov et al. (2007) and Zheng et al. (2015) obtained statistically significant results ($p$<0.03 and $p$<0.001): Morozov et al. observed a higher pregnancy rate within natural cycles, and Zheng et al. identified more positive outcomes when analyzing artificial cycles. The remaining selected studies did not show any statistical significance.
**Conclusion:** There is insufficient scientific evidence to state that the artificial cycle has better pregnancy rates than the natural cycle in women submitted to frozen embryo transfer. Limitations of the study included small number of articles and heterogeneity among the works.

### P-36. The effect of thyroid dysfunction on embryo quality among infertile women undergoing IVF/ICSI treatment

**Y.A.R. de Lima[1], T.M. da Silva[1], M.C. Silva e Maia[1], M.S. Ramos[1], M.Z.P. Brito[1], E.N. Santos[1], F.C. Approbato[1], K.Q. Poletto[1], M.S. Approbato[1]**

**Objective:** Thyroid dysfunction has been previously associated with infertility; however controversial evidence associates it with IVF outcomes. This study aimed to evaluate the effect of different TSH levels on embryo fragmentation at day 3.
**Methods:** A case-control study was performed. Patients attending an IVF laboratory were divided into three groups: group 1 (TSH levels 0.3-2.5mUI/L); group 2 (TSH levels 2.5-4.5mUI/L) and group 3 (TSH levels >4.5mUI/L). All groups were matched by age, FSH levels and absence of masculine factor. Risk (Odds Ratio - OR) of thyroid dysfunction to generate fragmented embryos on day 3 was calculated by Chi-square test using Epi Info software.
**Results:** A total of 224 cases were retrieved from medical files. Mean age among patients was 34.3 years (SD±3.7). Group 1 (euthyroidism) was composed by 172 patients (76.8%; CI95% 70.7-82.1%); Group 2 (subclinical hypothyroidism) had 45 patients (20.1%; CI95% 15.1-26.0%) and Group 3 (hypothyroidism) was formed by 7 patients (3.1%; CI95% 1.3-6.3%). No significant difference was observed among both groups regarding the number of classified embryos in day 3. Additionally, groups presenting thyroid dysfunctions had similar risk for embryo fragmentation than group with euthyroidism (OR=0.763; CI95%: 0.458-1.271; $p$=0.3615).
**Conclusion:** Although thyroid dysfunction groups were not associated with increased risk for embryo fragmentation on day 3, further studies should be performed in order to evaluate other IVF outcomes.

### P-37. Single versus double transfer of embryos in blastocysts in patients with good prognosis

**E. Araújo Filho[1], L.P. de Araújo[1], B.R. de Marchi[1], C.L. Fácio[1], L.A. Machado-Paula[1], L.F. Previato[1]**

**Objective:** To compare the results of single and double transfer of embryos of the 5th day (Blastocyst) in patients up to 39 years of age regarding the pregnancy rate, abortion, live births and multiple pregnancy.
**Methods:** A retrospective case-control study was performed with 165 patients from July 2015 to December 2017. Patients were divided into 2 groups: group 1 with 18 patients whose transfer was only 1 blastocyst and group 2 with 145 patients in which 2 blastocysts were transferred. Patients up to 39 years old were selected. The ovarian stimulation protocol was performed with recombinant FSH 150 IU from day 2 of the cycle and urinary human menopausal gonadotrophin ranging from 75 to 150 IU per day. Pituitary block was performed with GnRH antagonist when the largest follicle reached 15mm in diameter until the eve of the ovarian puncture. When at least 2 follicles/ovary reached 18mm of mean diameter, recombinant hCG (250mcg) was administered and ovarian puncture was performed 36 hours later. In these 2 groups were included only patients with embryos transferred on day 5. All embryo transfers were easy. Exclusion criteria: patients ≥40 years, PGD cases. For the statistical analysis chi-square was used.
**Results:** Of the 18 patients in group 1 who transferred 1 blastocyst, 10 became clinically pregnant (pregnancy rate: 55%) (positive embryonic heartbeat) and only 2 had abortion (20%). Of the 145 patients in group 2 who transferred 2 blastocysts, 89 became clinically pregnant (pregnancy rate: 61%) and 20 had abortion (abortion rate: 22%). In group 1 the multiple pregnancy rate was 10% (1 twin pregnancy). In group 2 the multiple pregnancy rate was 36%; 32 pregnant had twins ($p$<0.05%). The implantation rate in group 1 was 55% and in group 2 it was 42.7%. There was no statistical difference in the clinical pregnancy rate between groups 1 and 2 ($p$>0.05%). When we selected only the patients up to 35 years of age from the 2 groups (1 and 2), the pregnancy rate in group 1 was 53.3% (8 of 15 pregnancies) and in group 2 the pregnancy rate was 62.3% (66 of 106 pregnancies). Again, the difference was not statistically significant ($p$>0.05%). Only the multiple pregnancy rate was statistically higher in group 2 where we transferred 2 blastocysts ($p$<0.05%).
**Conclusion:** The number of patients in this study is small, especially in group 1, but the result indicates that for a well-selected group of patients, the single embryo transfer does not affect the IVF pregnancy rate. A prospective study is underway detailing the type of blastocyst transferred and comparing single and double transference to know, in our reality, whether there are or not differences in IVF outcome in these two groups.

### P-38. Validation of time-lapse incubator (Embryoscope, Vitrolife) in Brazilian IVF-lab routine

**M.M. Piccolomini[1], M. Nicolielo[1], A.R. Lorenzon[1], P.C. Serafini[1,2], E.L.A. Motta[1,3], J.R. Alegretti[1]**

**Objective:** Morphological characteristics have been the standard technique for embryo selection in IVF lab. Recently, the development of time-lapse incubators started to change the way embryos are evaluated and selected for transfer - before based in particular time-points and now based in continuous observation of embryo development. Besides that, the unchanged environmental condition for embryo development and evaluation is definitely a more stable and controlled condition. Our objective is to validate a time-lapse incubator in an IVF lab, comparing with a standard incubator system.

**Methods:** During a period of 6 months (between October 2017 and March 2018), we have selected 75 cycles, from patients undergoing IVF procedures at Huntington Medicina Reprodutiva (São Paulo, Brazil). It was selected patients with at least 8 mature oocytes (MII) and oocytes from the same patient were split between time-lapse (ES, Embryoscope) and K-system (KS) incubators (a total of 812 MII oocytes, 412 for ES and 391 for KS). Oocytes proceeded to ICSI (N=71) or IVF (n=4), according to lab indication, and all embryos were culture in the same supplemented media (CSCM, Irvine) until blastocyst stage (Day 5 or 6). Embryos cultured in KS were evaluated in day 1, day 3 and day 5 and/or 6 - embryo dishes were taken off the incubator for microscope evaluation - while ES embryos were evaluated at the same time of these days but maintaining the embryos inside the incubator, using the image system. When requested, embryo biopsy were hold on day 5 or 6 for all embryos from the same cycle.

**Results:** The average age of patients were 36.9 (±3.55) years old. The average number of oocytes proceeded to ICSI were 5.63±2.19 for ES and 5.20±2.19 for KS. The oocytes that proceeded to IVF the average number was similar (5.25±2,22 vs 5.00±2.31). Top quality day 3 embryos, blastocyst formation and top blastocyst rates were similar between incubators (64%±0.27 vs. 56%±0.37, $p$=0.07; 55%±0.32 vs. 58%±0.39, $p$=0.5; 55%±0.38 vs 47%±0.43, $p$=0.2).

**Conclusion:** In the terms of embryo quality and blastocyst formation rate, comparing the performance between two incubators systems - using the same patient oocytes cycle and same embryo media - there is no difference between ES and KS, which is an important parameter to consider in an IVF lab before changing routine. The potential benefit of time-lapse incubator in other parameters, such as live birth rates, remain to be elucidated.

### P-39. Vitrification with artificial collapse: is there a difference in embryo survival and gestation rate?

**R. Azambuja[1], M.R. Hentschke[1], L. Okada[1], V.P. Reig[1], A. Tagliani-Ribeiro[1], S. Flach[1], L. Proença[1], J. Alexandria[1], M. Badalotti[1], A. Petracco**

*[1]Fertilitat - Centro de Medicina Reprodutiva.*

**Objective:** Blastocoel is filled with large amounts of fluid during process of vitrification, which may cause intracellular ice-crystal formation, a potentially lethal damage of the embryo during cooling, influencing the dehydration shrinkage rate. Some studies suggest that collapsing the embryo prior to vitrification can reduce injuries and increase the thawed blastocyst survival and pregnancy rate. The objective of this study was to determine the effect of artificial shrinkage on D5/D6 blastocysts by laser-induced collapse before vitrification on the embryo survival and pregnancy rate.

**Methods:** A retrospective, case-control study was performed with a total of 292 cycles using frozen/thawed embryos which were analyzed at Fertilitat - Centro de Medicina Reprodutiva - in Southern Brazil, from 2013 to 2017. Before vitrification, the blastocoelic cavity was collapsed by a laser pulse (500µs) on D5 (n=42) or D6 (n=116) embryos. Some blastocysts were vitrified without collapsing and were used as controls (n=134). Vitrification and thawing were performed according to Kitazato´s protocol (Kuwayama *et al*., 1998). The variables were analyzed using the Chi-square test.

**Results:** All groups (collapsed D5, collapsed D6, and controls) presented a higher survival rate: 98%, 96%, and 92% respectively. No statistically significant result was found in embryo survival or pregnancy rate between groups ($p$>0.05). Pregnancy rate ranged from 50.7% (controls) to 54.8% (D5).

**Conclusion:** No significant difference was found between collapsed and non-collapsed blastocysts regardless the day of vitrification (D5 or D6) compared to controls Therefore, artificial shrinkage does not seem to be an effective tool to improve pregnancy rate. However, in some centers, the embryo quality after thaw convinced the researchers to recognize the clinical benefit of artificial shrinkage and to implement this technique in vitrification practice.



P01. Transferring a morula embryo can interfere in implantation of a blastocyst?

Lopes V.M.
Brasileiro J.P.B.
Lopes J.R.C.
Lacerda T.S.
Rocha C.A. da
Oliveira S.A. de
Pereira P.C.M.
Instituto Verhum - Brasília, DF; Brazil.
CENAFERT- Salvador, BA; Brazil.

P-02. Amino acids supplementation in culture of cumulus-oocyte complexes: does it matter?

Kulmann M.I.R.
Martello C.
Bos-Mikich A.
Pagnoncelli N.
Frantz G.
Dutra C.
Frantz N.
Centro de Reprodução Humana Nilo Frantz, IVF Laboratory, Porto Alegre, Brazil.
Universidade Federal do Rio Grande do Sul, Basic Sciences and Health Institute, Porto Alegre, Brazil.
Centro de Reprodução Humana Nilo Frantz, Clinical Department, Porto Alegre, Brazil.

P-03. Quantitative and qualitative analysis of microorganisms in an Assisted Reproduction laboratory

Poletto K.Q. 1
Lima Y.A.R. de 1
Rezende D.F. 1
Castro E.C. de 1
Approbato M.S. 1
1 Laboratório de Reprodução Humana, Hospital das Clínicas, Universidade Federal de Goiás - UFG, Goiânia, GO, Brazil.

P-04. Aneuploidy screening data in Brazilian population - 4.5 years of PGS/PGT-A in IVF-lab routine

Reis I. 1
Romanenghi G. 1
Barros B. 1
Lorenzon A.R. 1
Chehin M.B. 1
1 Huntington Medicina Reprodutiva, São Paulo, SP, Brazil.

P-05. Anogenital distance is associated with semen quality and serum reproductive hormones in brazilian young men

Procópio M.S. 1
Borges D.C. 1
Fernandes C.G. 1
Santos M.L. dos 1
Leite R.A. 1
Lacerda S.M.S.N. 1
França L.R. de 1
Reis A.B. 2
1 Department of Morphology. Institute of Biological Sciences, Federal University of Minas Gerais, Belo Horizonte, MG, Brazil.
2 Department of Surgery. Medicine Faculty, Federal University of Minas Gerais, Belo Horizonte, MG, Brazil.

P-06. Evaluation of the influence of the number of retrieved oocytes on the success of ICSI

Polisseni J. 1
Assunção C.M. de 1
Polisseni F. 1
Rosa J.V. 1
Coutinho L.M. 1
1 Clínica Nidus Medicina Reprodutiva.

P-07. Evaluation of seminal parameters after freezing in two types of Brazilian cryoprobes: with or without egg yolk low-density lipoprotein

Bossi R.L. 1
Villa B.C. 2
Sampaio M.A.C. 2
França P.P. 1
Alvarenga D.M. 1
Geber S. 1
1 Centro de Medicina Reprodutiva - ORIGEN.
2 Centro Universitário UNA.

P-08. Clinical outcome of in vitro maturation treatment in a series of patients with polycystic ovaries and polycystic ovaries syndrome

Bos-Mikich A. 1
Kulmann M.I.R. 2
Oliveira N.P. de 2
Gross C. 2
Toson B. 2
Ferreira M. 2
Frantz N. 2
1 Universidade Federal do Rio Grande do Sul.
2 Nilo Frantz Medicina Reprodutiva.

P-09. Correlation between number full-term pregnancies after ICSI treatment and day of embryo transfer

Polisseni J. 1
Assunção C.M. de 1
Polisseni F. 1
Rosa J.V. 1
Coutinho L.M. 1
1 Clínica Nidus Medicina Reprodutiva.

P-10. Clinical data of patients who cryopreserved spermatozoa at a private clinic in Curitiba/PR due to future gonadotoxic treatments

Scaraboto D. 1
Centa L.J.R. 1
Saruhashi I.T. 1
1 ANDROLAB - Clínica e Laboratório de Reprodução Humana e Andrologia.

P-11. Delayed intracytoplasmic sperm injection: is it worth it?

Braga D.P.A.F. 1 2
Iaconelli C.A.R. 1
Brogilato C.C.V. 1
Zanetti B.F. 1 2
Setti A.S. 1 2
Iaconelli Jr. A. 1 2
Borges Jr. E. 1 2
Ferreira R.C. 1
1 Fertility Medical Group, São Paulo, SP - Brazil.
2 Instituto Sapientiae - Centro de Estudos e Pesquisa em Reprodução Assistida, São Paulo, SP - Brazil.

P-12. Difficulties in homoaffective couples' IVF

Bartmann A.K.
Silva L.F.I. da
Franco A.L.C.
Moriwake K.T.
Brioni L.F.
1 Centro de Reprodução Humana - clínica ana bartmann.
2 Universidade de Ribeirão Preto (UNAERP).

P-13. Does endometrioma surgery affect pregnancy rates in women with intestinal endometriosis?

Carneiro M.M.
Amorim L.C.V.
Ávila I. de
Costa L.M.P.
Machado L.G.R.
Ferreira M.C.F.
Equipe Multidisciplinar de Endometriose do Hospitla Biocor.
Departamento de Ginecologia e Obstetrícia e Obstetrícia da Faculdade de Medicina da UFMG.

P-14. Endometriosis III and IV as a risk factor for tubal obstruction among infertile women

Approbato F.C. 1
Approbato M.S. 1
Rezende D.F. 1
Ramos M.S. 1
Santos E.N. 1
Brito M.Z.P. 1
Silva T.M. 1
Maia M.C.S. 1
Lima Y.A.R. 1
Moraes A.V.S. 1
Castro E.C. 1
Rosilho K.Q.P. 1
Sasaki R.S. 1
Finotti M.C.F. 1
1 Reproduction Human Laboratory. Obstetrics and Gynecology Dept. Federal University of Goias State, Brazil.

P-15. Tubal factor etiologies in infertile patients from a public assisted reproductive service

Rezende D.F. 1
Lima Y.A.R. de 1
Poletto K.Q. 1
Approbato M.S. 1
Castro E.C. de 1
Moraes A.V.S. 1
1 Laboratório de Reprodução Humana, Hospital das Clínicas, Universidade Federal de Goiás - UFG, Goiânia, GO.

P-16. Expression of the Luteinizing Hormone Receptor and its isoforms in animal model for Poor Responder Phenotype

Wohlres-Viana S.
Arashiro E.K.N.
Siqueira L.G.B.
Machado M.A.
Viana J.H.M.
Universidade Federal de Juiz de Fora.
Universidade Federal Fluminense.
Embrapa Gado de Leite.
Embrapa Recursos Genéticos e Biotecnologia.

P-17. Impact of cryopreservation of spermatozoa from PESA and TESE on the prognosis of ICSI. Outcomes from a multicenter study

Pereira T.R. 1
Lopes V.M. 1
Brasileiro J.P.B. 1
Tierno N.Z. 1
Lacerda T.S. 1
Rocha C.A. da 1
Maciel J.J.M. 2
1 Instituto Verhum - Brasília, DF.
2 CENAFERT - Salvador, BA.

P-18. Incidence of acquired thrombophilia in Assisted Reproduction

Araújo Filho E. 1
Araújo L.P. de 1
Marchi B.R. de 1
Fácio C.L. 1
Machado-Paula L.A. 1
Previato L.F. 1
1 Centro de Reprodução Humana de São José do Preto (CRH Rio Preto), São José do Rio Preto, SP, Brasil.

P-19. Influence of endometrial type on success in Assisted Reproduction Techniques

Polisseni J.
Assunção C.M. de
Polisseni F.
Rosa J.V.
Coutinho L.M.
Rodrigues V.O.
Clínica Nidus Medicina Reprodutiva.
Universidade Federal de Juiz de Fora.

P-20. IVF/ICSI. Pregnancy chance by morphologic classification and number of D4 transferred morulae

Rocha J.P. 1
Batista M.P. 1
Borges G.C. 1
Castro E.C. de 1
Castro C.L.A. de 1
Oliveira V.A. de 1
Florencio R.S. 1
1 Humana Medicina Reprodutiva - Goiânia/GO.

P-21. IVF/ICSI. Chemical, clinical, and ongoing pregnancy rate of top embryos transferring D4 versus D5-6

Florencio R.S. 1
Rocha M.N.C. 1
Rocha J.P. 1
Borges G.C. 1
Borges M.R. 1
Finotti M.C.C.F. 1
Santos F.C. 1
1 Humana Medicina Reprodutiva - Goiânia/GO.

P-22. Live birth after delayed intracytoplasmic sperm injection of immature oocyte- a case report

Braga D.P.A.F. 1 2
Ferreira R.C. 1
Brogilato C.C.V. 2
Zanetti B.F. 2
Setti A.S. 1 2
Iaconelli Jr. A. 1 2
Borges Jr. E. 1 2
Iaconelli C.A.R. 1
1 Fertility Medical Group, São Paulo, SP - Brazil.
2 Instituto Sapientiae - Centro de Estudos e Pesquisa em Reprodução Assistida, São Paulo, SP - Brazil.

P-23. Identified microorganisms on clean room surface samples from an Assisted Reproduction laboratory

Poletto K.Q. 1
Lima Y.A.R. de 1
Rezende D.F. 1
Castro E.C. de 1
Approbato M.S. 1
1 Laboratório de Reprodução Humana, Hospital das Clínicas, Universidade Federal de Goiás - UFG, Goiânia, GO.

P-24. Otimizing medical consultation time in assisted reproduction

D'Avila A.M. 1
Bianchi L.C. 1
1 Embrios centro de Reprodução Humana.

P-25. Ovarian vitrification imply in gene expression changes related with ultrastructural adaptations that maintain the ovarian and oocyte viability

Pereira L.A.A.C. 1
Segatelli T.M. 2
Jorge E.C. 2
Campos-Junior P.H.A. 1
1 Fertility Preservation Research Group, Universidade Federal de São João del Rei.
2 Laboratório de Biologia Oral e do Desenvolvimento, Universidade Federal de Minas Gerais.

P-26. Seminal analysis in patients with normal and altered BMI from an Assisted Reproduction clinic of Curitiba, Paraná

Scaraboto D. 1
Cho R.Y. 1
Saruhashi I.T. 1
Centa L.J.R. 1
1 ANDROLAB - Clínica e Laboratório de Reprodução Humana e Andrologia.

P-27. Semen analysis patterns in patients from an assisted reproduction clinic in Curitiba, Paraná

Scaraboto D. 1
Saruhashi I.T. 1
Cho R.Y. 1
Centa L.J.R. 1
1 ANDROLAB - Clínica e Laboratório de Reprodução Humana e Andrologia.

P-28. Patients' profile of social egg freezing in a Private Center in São Paulo, Brazil: evaluation of 5 years of experience

Cavagnoli M. 1
Gomes C. 1
Lorenzon A.R. 1
Serafini P.C. 1 2
Motta E.L.A. 1 3
1 Huntington Medicina Reprodutiva, São Paulo, SP, Brazil.
2 Departamento de Ginecologia e Obstetrícia, Faculdade de Medicina da Universidade de São Paulo, São Paulo, SP, Brazil.
3 Departamento de Ginecologia, Escola Paulista de Medicina, Universidade Federal de São Paulo, São Paulo, SP, Brazil.

P-29. Cytogenetic profile of the infertile couples evaluated by human reproduction genetics at the Hospital Materno Infantil de Brasilia

Medina C.T.N. 1
Reis B.F. dos 1
Crispin A.A. 1
Antonialli G.P.M. 1
Leite A.C.R. 1
Versiani B.R. 1
Silva E.C.N. da 1
Couto S.M. do 1
Vasconcelos G.R. 1
Oliveira T.M. de 1
Cardoso M.T.O. 1
Costa R.R. 1
Pimental E. 1
Zavattiero N. 1
1 Secretaria de Estado de Saúde do Distrito Federal.

P-30. Preservation of fertility in women submitted to gonadotoxic treatments in the public health system of Minas Gerais

Rodrigues J.K. 1 2
Monteiro C.S. 1
Silva T.E. 1
Canabrava C.M. 1
Cruzeiro I.K.D.C. 1 3
Pereira F.A.N. 1 3
Reis F.M. 1
1 Universidade Federal de Minas Gerais.
2 In Vitro Embriologia Clínica e Consultoria.
3 Life Search Serviço de Reprodução Humana.

P-31. Prevalence of subclinical hypothyroidism in infertile patients

Rezende D.F. 1
Lima Y.A.R. de 1
Poletto K.Q. 1
Approbato M.S. 1
Castro E.C. de 1
Moraes A.V.S. 1
1 Laboratório de Reprodução Humana, Hospital das Clínicas, Universidade Federal de Goiás - UFG, Goiânia, GO.

P-32. Nursing care protocol to the patient submitted to Assisted Reproductive Technique - Safe Surgery

Righetti E.A.V. 1
Gonçalves A.F. 1
Magrin S.F.F. 1
Luz M.P. da 1
Resende S.S. 2
Maziero V.G. 1
1 Hospital Universitário Maria Aparecida Pedrossian - HUMAP/Universidade Federal de Mato Grosso do Sul - UFMS.
2 Centro de Reprodução Humana Doutora Suely Resende.

P-33. Case Report: Recovery of testicular function after prolactinoma treatment

Macedo J.F. de
Oliveira M.R.
Gomes L.M.O.
Macedo G.C. de
Macedo G.C. de
Gomes D.O.
Maciel L.C.
Clínica Reproferty - São José dos Campos - SP - Brazil.
Universidade de Taubaté - Taubaté - SP - Brazil.

P-34. Assisted Human Reproduction under the public healthcare system: implementation of comprehensive care policies and the current scenario

Garcia R.S.
Moraes R.A. de
Turci M.A.
Universidade Federal de Viçosa; Universidade José do Rosário Vellano - Unifenas.
Universidade José do Rosário Vellano - Unifenas.

P-35. Pregnancy rates between natural and artificial cycles of women submitted to frozen embryo transfer: a meta-analysis

Poletto K.Q. 1
Lobo M.P. 1
Giovanucci M. 1
Lima Y.A.R. de 1
Approbato M.S. 1
Castro E.C. de 1
1 Laboratório de Reprodução Humana, Hospital das Clínicas, Universidade Federal de Goiás - UFG, Goiânia, GO.

P-36. The effect of thyroid dysfunction on embryo quality among infertile women undergoing IVF/ICSI treatment

Lima Y.A.R. de 1
Silva T.M. da 1
Maia M.C. Silva e 1
Ramos M.S. 1
Brito M.Z.P. 1
Santos E.N. 1
Approbato F.C. 1
Poletto K.Q. 1
Approbato M.S. 1
1 Laboratório de Reprodução Humana, Hospital das Clínicas, Universidade Federal de Goiás - UFG, Goiânia, GO.

P-37. Single versus double transfer of embryos in blastocysts in patients with good prognosis

Araújo Filho E. 1
Araújo L.P. de 1
Marchi B.R. de 1
Fácio C.L. 1
Machado-Paula L.A. 1
Previato L.F. 1
1 Centro de Reprodução Humana de São José do Preto (CRH Rio Preto), São José do Rio Preto, SP, Brasil.

P-38. Validation of time-lapse incubator (Embryoscope, Vitrolife) in Brazilian IVF-lab routine

Piccolomini M.M. 1
Nicolielo M. 1
Lorenzon A.R. 1
Serafini P.C. 1 2
Motta E.L.A. 1 3
Alegretti J.R. 1
1 Huntington Medicina Reprodutiva, São Paulo, SP, Brazil.
2 Departamento de Ginecologia e Obstetrícia, Faculdade de Medicina da Universidade de São Paulo, São Paulo, SP, Brazil.
3 Departamento de Ginecologia, Escola Paulista de Medicina, Universidade Federal de São Paulo, São Paulo, SP, Brazil.

P-39. Vitrification with artificial collapse: is there a difference in embryo survival and gestation rate?

Azambuja R. 1
Hentschke M.R. 1
Okada L. 1
Reig V.P. 1
Tagliani-Ribeiro A. 1
Flach S. 1
Proença L. 1
Alexandria J. 1
Badalotti M. 1
Petracco A.
1 Fertilitat - Centro de Medicina Reprodutiva.

